# The Urban Built Environment, Walking and Mental Health Outcomes Among Older Adults: A Pilot Study

**DOI:** 10.3389/fpubh.2020.575946

**Published:** 2020-09-23

**Authors:** Jenny Roe, Andrew Mondschein, Chris Neale, Laura Barnes, Medhi Boukhechba, Stephanie Lopez

**Affiliations:** ^1^Center for Design & Health, School of Architecture, University of Virginia, Charlottesville, VA, United States; ^2^Department of Urban and Environmental Planning, School of Architecture, University of Virginia, Charlottesville, VA, United States; ^3^Engineering Systems and Environment Department, School of Engineering, University of Virginia, Charlottesville, VA, United States

**Keywords:** cognitive health, stress, air pollution, noise pollution, urban green space, wearable sensors

## Abstract

The benefits of walking in older age include improved cognitive health (e.g., mental alertness, improved memory functioning) and a reduced risk of stress, depression and dementia. However, research capturing the benefits of walking among older people in real-time as they navigate their world is currently very limited. This study explores cognitive health and well-being outcomes in older people as they walk in their local neighborhood environment. Residents from an independent living facility for older people (mean age 65, *n* = 11) walked from their home in two dichotomous settings, selected on the basis of significantly different infrastructure, varying levels of noise, traffic and percentage of green space. Employing a repeated-measures, cross over design, participants were randomly allocated to one of two groups, and walked on different days in an urban busy “gray” district (a busy, built up commercial street) vs. an urban quiet “green” district (a quiet residential area with front gardens and street trees). Our study captured real-time air quality and noise data using hand-held Airbeam sensors and physiologic health data using a smart watch to capture heart rate variability (a biomarker of stress). Cognitive health outcome measures were a pre- and post-walk short cognitive reaction time (SRT) test and memory recall of the route walked (captured via a drawn mental map). Emotional well-being outcomes were a pre- and post-walk mood scale capturing perceived stress, happiness and arousal levels. Findings showed significant positive health benefits from walking in the urban green district on emotional well-being (happiness levels) and stress physiology (*p* < 0.05), accompanied by faster cognitive reaction times post-walk, albeit not statistically significant in this small sample. Cognitive recall of the route varied between urban gray and urban green conditions, as participants were more likely to rely on natural features to define their routes when present. The environmental and physiologic data sets were converged to show a significant effect of ambient noise and urban conditions on stress activation as measured by heart rate variability. Findings are discussed in relation to the complexity of combining real-time environmental and physiologic data and the implications for follow-on studies. Overall, our study demonstrates the viability of using older people as citizen scientists in the capture of environmental and physiologic stress data and establishes a new protocol for exploring relationships between the built environment and cognitive health in older people.

## Introduction

Exercise is extremely important to healthy aging, reducing the risk of cardio-vascular disease, susceptibly to stress and depression, and improving cognitive functioning. Physical activity is an important modifiable risk factor for reducing the risk of dementia and cognitive decline in older age (1, 2). Exercise also has a direct role in brain health; even light exercise in older adults (55–80 years) can increase the volume of the anterior hippocampus (3), a key part of the brain network that supports spatial memory (our memories of place and spatial relations). In the same age group, research has shown 30 min of exercise may increase neural processes underlying semantic memory activation (our recall of objective knowledge) in healthy older adults (4). All of these types of memories facilitate our experience of space but with aging, this memory network – particularly semantic memory – can diminish and become disrupted with older age, as well as with certain forms of dementia. But, regular physical activity can help maintain memory performance by increasing neural efficiency (5) and is associated with increased white-matter volume in older people (which allows for communication between different brain regions) and brain plasticity (the brain's ability to adapt to changes in the environment or new situations) [reported in Macpherson et al. (6)].

Exercise also plays a role in supporting social interrelations. Older people have a heightened risk of social isolation and loneliness; walkable neighborhoods and using different modes of transport (bicycle, public transport) can significantly reduce loneliness in older people (7) offering impromptu opportunities for social interaction. But only between 6 and 29.8 per cent of older adults aged 65 plus attain the US recommended activity guidelines (i.e., ≥150 min of moderate-intensity equivalent minutes of activity per week in bouts of at least 10 min) (8) for “lifestyle” and “ambulatory” activities, respectively (9).

Older people are often hampered from walking in their local neighborhood owing to traffic, noise, air pollution and poorly maintained sidewalks. In addition, people living in lower income neighborhoods are less likely to encounter features that encourage walking, including street trees and parks. Being physically active in older age is now identified as one simple, low cost strategy that can help reduce the burden of dementia, the greatest global challenge for health and social care in the 21st century (10). How we design the environment to promote walkability therefore has an important role to play in healthy aging.

Improving walkability includes regulating for and reducing air and noise pollution. A systematic review of longitudinal cohort studies (*n* = 13) identified an association between greater exposure to airborne pollutants and an increased risk of dementia and cognitive decline (11). Increases in dementia risk were found for fine particulate (PM_2.5_), nitrogen oxides (NO_2_/NO_x_) and carbon monoxide (CO). One study showed people aged 50 plus living with high levels of air pollutants have a 40 percent greater risk of developing dementia as compared to those living with lower air pollution (12). Because traffic is a large source of many different pollutants, concentrations of air pollutants are often elevated near roadways (13, 14) with residential proximity to roadways linked to a variety of adverse health effects [e.g., (15, 16)]. The contribution of noise to this association has rarely been examined and findings to date are inconclusive. However, excessive noise is associated with physical and mental illness and with higher levels of heart disease, stress, poor sleep quality and cognitive impairment (17). Despite the evidence that excessive urban noise can contribute to negative health outcomes, governments rarely regulate average or ambient street noise (18). Instead, local governments primarily regulate noise with regard to individual instances, such as a single vehicle's engine or horn noise. Using our pilot study's location as an example, Richmond, Virginia sets the maximum allowable dBA of a vehicle, measured at a distance of at least 50 feet (15 m) at 86dBA on streets with a speed limit of 35 MPH (56 kph) or less (19). However, this regulatory standard would have little effect on average street noise levels. While not available for Richmond, a 2015 study of NYC street noise found a mean street noise level of 73.4dBA, with the busiest streets ranging up to 95.0 dBA on a typical day (18).

Decades of research have shown that exposure to natural environments, or green space, can act as an equalizer in health inequities, such as cardiovascular disease (CVD), obesity, psychological well-being, stress regulation and social health across the lifespan (20). Low-income neighborhoods with a high proportion of older residents are disproportionately healthier if their neighborhoods contain good quality, publicly accessible green space (21). Living in areas with walkable green space is associated with increased life longevity in older urban citizens (22, 23). Furthermore, walking in urban green space (“green exercise”) - as compared to urban busy districts - is associated with improved emotional well-being and mental alertness (24, 25).

Theoretically, it's postulated that one of the mechanisms by which green space delivers these health benefits is through the air pollution pathway (i.e., trees and other greenery filter pollutants such as PM_2.5_ improving respiratory health and reducing the risk of heart disease); other postulated pathways include the effect of green space on stress regulatory mechanisms (e.g., reduced allostatic load, the neuroendocrine system), the increased likelihood of exercising in green spaces (owing to improved place aesthetics) as well as the social benefits that accrue from meeting people in green spaces (either on an impromptu or organized basis) (26). It is posited that relationships between urban green space and health outcomes are stronger in older people because they spend more time in their residential environment owing to retirement or limited mobility.

But green space is not distributed equitably; economically deprived areas often contain both lower quantity and poorer quality of green space resulting in less opportunity for green exercise and recreation (27), and in turn, fewer opportunities for public health.

Recently, the evidence of positive health effects has grown through the application of biologic indicators to capture immediate physiologic changes that occur in response to exposure to the outdoors, aided by the advancement in mobile technologies (e.g., smart phones). This has resulted in captures of biologic responses to the outdoors including heart rate variability (HRV), blood pressure, saliva, actigraph, urine, and electroencephalography. These studies generally have small sample sizes, are mostly carried out in healthy (student) populations, are rarely carried out in older populations and have generated mixed results [see (28) for a systematic review].

Additionally, the effect of urban environments on individuals can be measured through its effects on spatial knowledge and cognitive maps. Cognitive mapping exercises, such as sketch mapping of traveled routes, can highlight the elements of the environment that are most salient to a traveler (29). Salient elements vary not just by route or environment, but also by socio-cultural factors, such as ethnicity (30). Older adults, in particular, may have difficulty forming cognitive maps after travel (31). However, the elements of the built environment that are most salient to older adults as they travel through urban environments remains little examined.

### Aims and Rationale

We recruited a sample of retired older people on lower-incomes (*n* = 11) to examine the feasibility of (a) integrating real-time physiological data with real-time environmental data; and (b) establish a new study protocol integrating cognitive health measures with real-time stress measures to explore outdoor exposure effects in an aging population.

Given the evidence above, we developed two hypotheses:

1. Older people, on a low income, will experience mental health benefits from walking in local neighborhoods that include urban street stress, and other urban natural features such as domestic gardens and nearby parks. Benefits will extend to subjective well-being, cognitive functioning (reaction times and spatial memory), and physiological indicators of stress.
2. Lower levels of air and noise pollution will result in improvements across the outcome measures described in Hypothesis 1.

We tested the above hypotheses using the mechanism of a walk in two distinctly dichotomous environmental settings with different spatial and environmental characteristics: an urban “gray” walk in a busy, trafficked urban district vs. an urban “green” walk in a quieter residential district with front gardens, street trees and a pocket park. Using a walk as the outdoor exposure mechanism replicates tried and tested protocol in environment-health research (24, 25, 32, 33).

## Methods

### Subjects

Participants were healthy adults (*n* = 11, mean age 64.8, 6 male: 5 female) living in an independent residential facility in Richmond, Virginia. Participants were recruited by purposive sampling methods to ensure they met the required inclusion criteria. We carried out a baseline health survey in a larger sample prior to identify fit, healthy adults capable of walking at ease (unassisted) for 15–20 min. Exclusion criteria for study participation included visual impairment, chronic mental illness and a history of epileptic or psychiatric disorders. All participants were required to be able to walk, unassisted by another person, for at least 15 min. Ethical approval for the study was provided by the University of Virginia Institutional Review Board for Health Sciences Research (IRB-HSR) with informed and signed consent a condition of taking part in the study.

### Study Design

**Figure 1:**
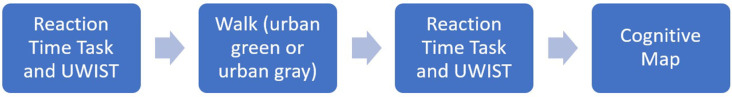
Experimental protocol for each testing day. Note: participants walk condition was counterbalanced between days.

We employed a repeated-measures, cross over design, ensuring participants act as their own control. Participants were randomly allocated to one of two groups, each of 5–6 participants: Group 1 walked the urban “gray” route on Day 1, followed by the urban “green” route on Day 2, and Group 2 vice versa, with a 1-day intervening period between walks (see Figure 1). The two dichotomous walk routes are described above.

### Walking Routes

The two walk routes were located nearby to participants' residential home and were selected on the basis of significantly different levels of green space and gray infrastructure (*z* = −18.578, *p* < 0.00001): the urban “gray” walk comprised 89% gray infrastructure and 11% green infrastructure, compared to the urban “green” walk which comprised 59% gray and 41% green infrastructure. See Table 1 for further breakdown by land use cover (and supplemental data for maps of the spatial data for each route). From hereon we refer to the walk routes as “urban gray” and “urban green.”

**Table 1:** Percentage landcover for “gray” and “green” walks.

**Landcover areas**	**Urban gray (%)**	**Urban green (%)**
Non-building impervious	74%	48%
Non-tree vegetation	2%	16%
Tree canopy	9%	25%
Building impervious	15%	11%

**Figure 2:**
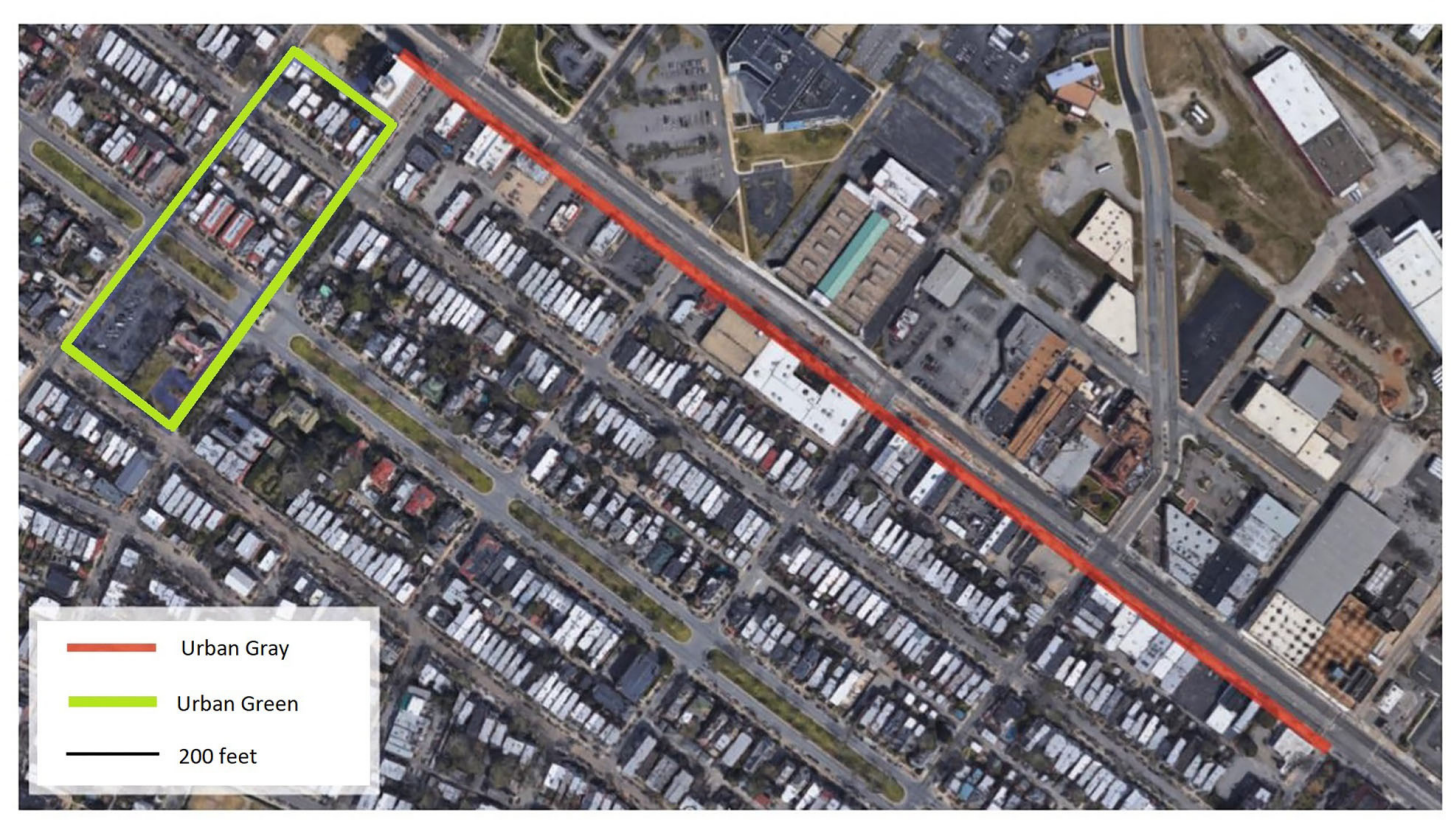
Aerial view of the two walking routes.

**Figure 3:**
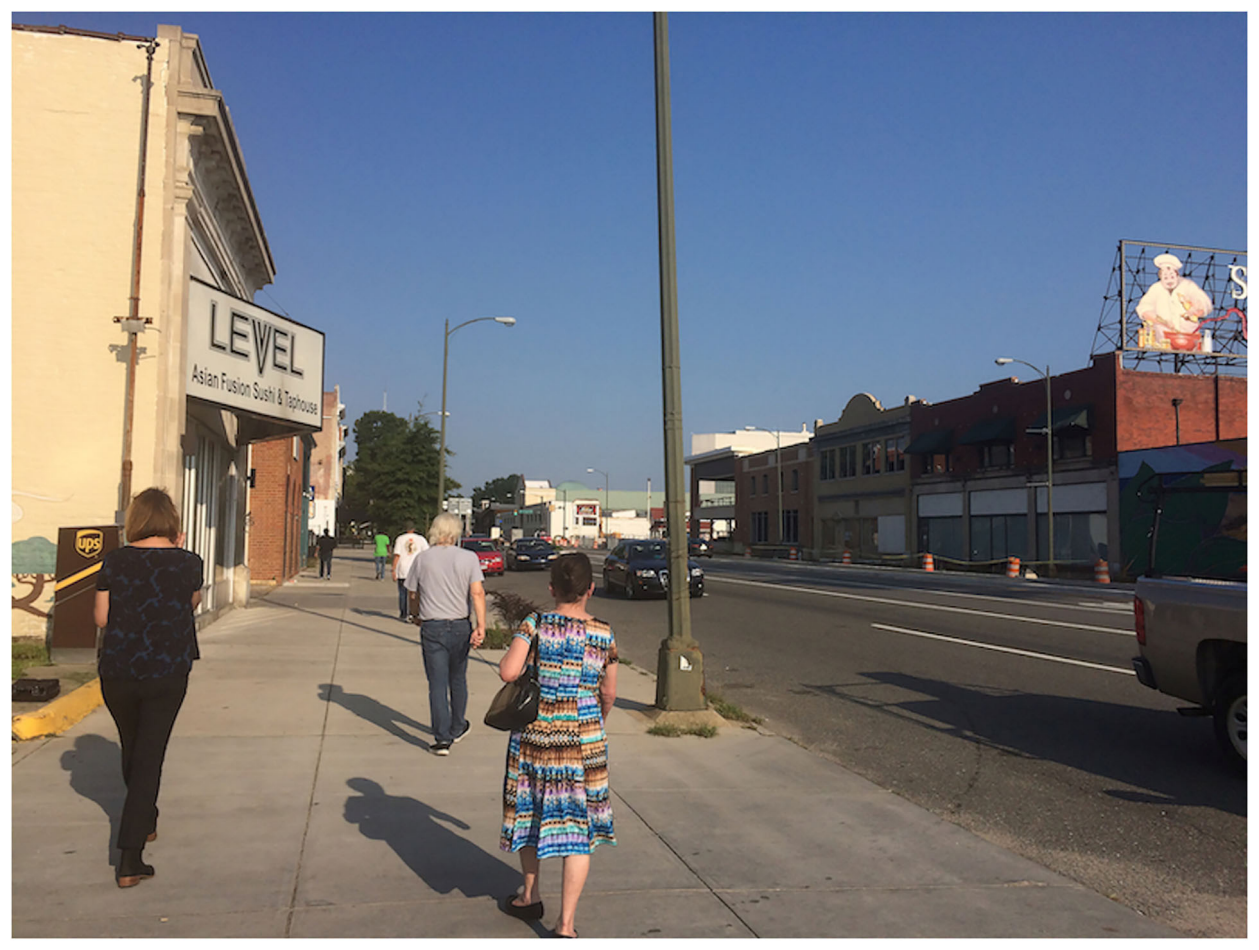
The urban busy “gray” walk.

**Figure 4:**
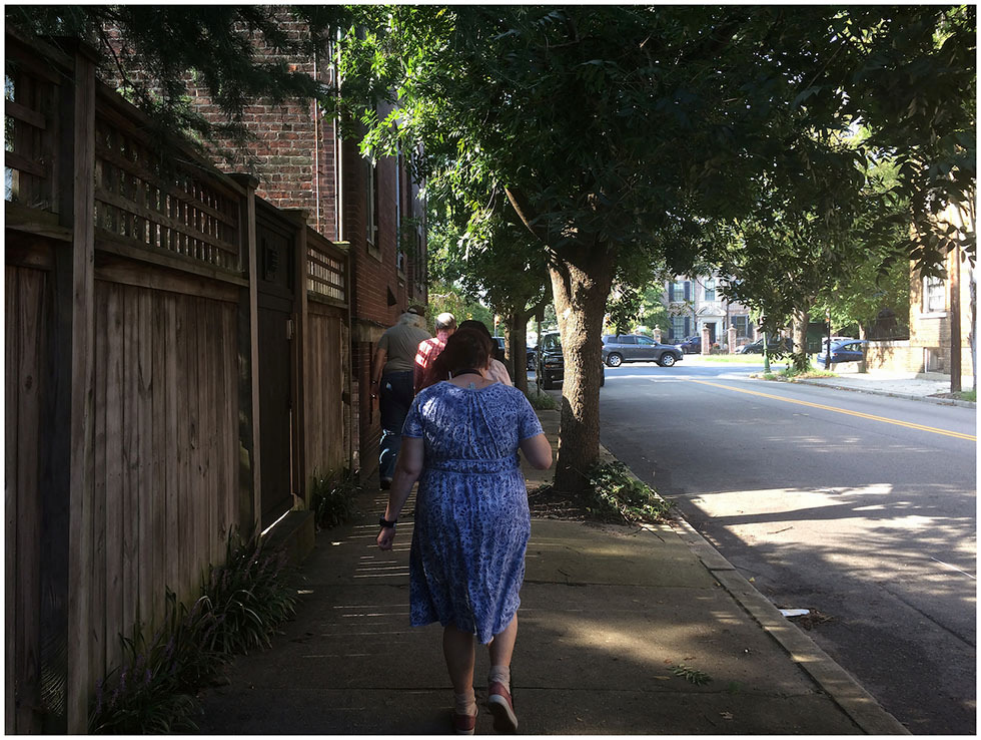
The urban quiet “green” walk.

The urban gray walk was characterized by a wide road system (4-lanes) with heavy traffic (including trucks and buses), a wide sidewalk with shops and restaurants fronting onto it, a flat gradient, and incorporated some minor road crossings enroute. It was linear in spatial composition (see Figure 2 below). The urban green walk was characterized by a narrower road system (two lanes), street trees, residential with front gardens, and included a small park, some historic buildings, and some road crossings. It was circular in spatial composition. See Figure 2 below for the two routes walked, and Figures 3, 4 for the visual context of the two settings. Participants walked at either 8:30 A.M. or 9:30 A.M. on Day 1 and 2 in small groups of 5-6. The walk routes were orientated in order to allow for safe road crossings. Participants were instructed not to eat, smoke, talk to each other or chat on their mobile phones. The weather condition on Day 1 was warm (temp 73F) and sunny; Day 2 was warm (temp 70F) and overcast with some occasional spots of rain.

Participant information about the study was provided prior to fieldwork, with signed consent checked by researchers prior to data collection. Data collection took place in a public meeting room at the residential facility on the day of the walk. Participants (living on-site) were asked to arrive 10 min prior to the walk-start time and completed a series of mental health tests and were fitted with a smart watch capturing heart rate (described below). They were asked to follow a walk leader and instructed not to eat, smoke, talk to each other or chat on their mobile phones during the walk. One of the research team led the walk, whilst another researcher walked at the tail-end to ensure participants did not encounter any difficulty. On returning to the residency, participants repeated the mental health tests (described below) and the smart watch was removed, with the data immediately backed up on a computer.

### Outcome Measures

#### Measures of Psychological Well-Being

We used the following psychological scales, previously used in senior populations.

1. **Mood** was measured using the short version of the University of Wales Institute of Science and Technology (UWIST) Mood Adjective Check List (MACL) (34, 35), giving acute measures of hedonic tone (valence), stress and (physical) arousal, shown as three individual scores. The hedonic tone scale measures overall pleasantness of mood, and is associated with feelings of somatic comfort and well-being, the stress scale measures feelings of subjective tension and the arousal scale measures feelings of subjective energy. Scores are obtained from summation of individual item scores pertaining to each of the three mood components.
2. **Subjective well-being** was measured using the short version of the Warwick and Edinburgh Mental Well-being Scale (SWEMWBS), a 7-item scale which measures how people have felt over a 2-week time scale (e.g., feeling relaxed, feeling useful), with responses rated on a 5-point Likert scale from “none of the time” to “all of the time.” Scores can range from 7 (indicating very low well-being) to 35 (very high well-being). This scale captures a longer-term subjective well-being and was employed to determine if participants' well-being was stable during the period of the experiment.

#### Measures of Cognitive Functioning

**Figure 5:**
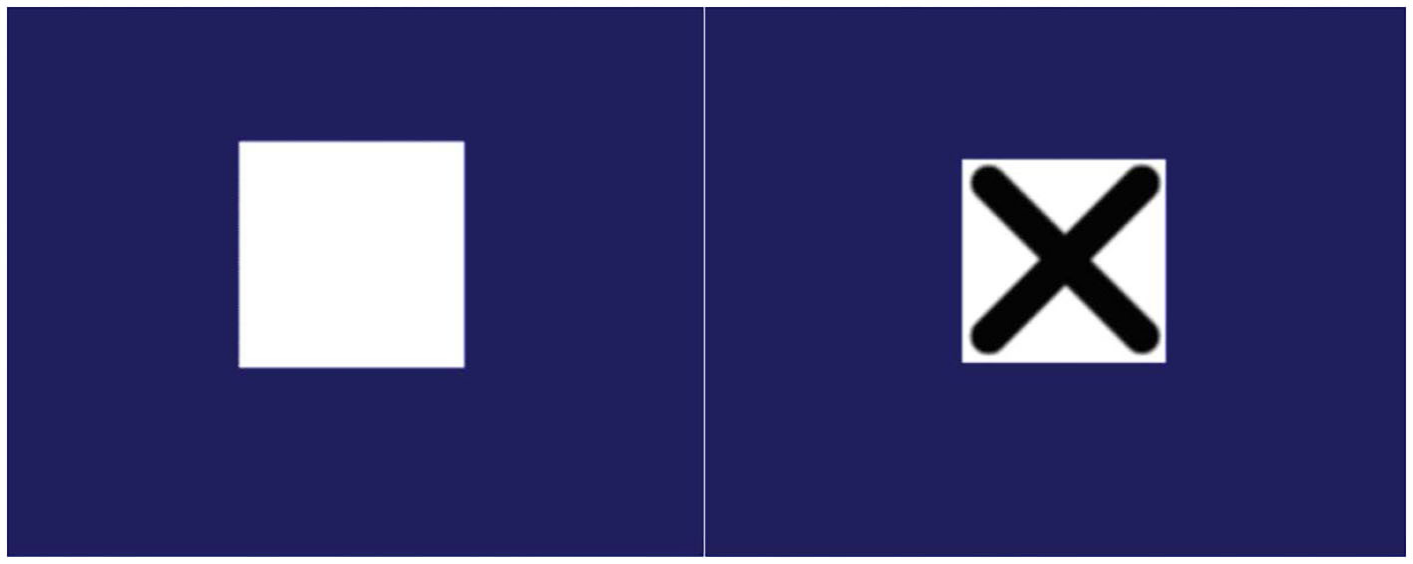
Screen shots of the SRT paradigm; participants respond when an X appears in a central white box.

**(1) Reaction time** was measured using the Deary-Liewald computer-based simple reaction time test (SRT) (36). In the SRT, participants press a key in response to a single stimulus (the appearance of a diagonal cross within a square) displayed on a computer screen (see Figure 5 below). Each time the cross appears, participants respond by pressing the response key as quickly as possible. Each cross remained on the screen until the key was pressed, after which it disappears, and another cross appeared some seconds later. The inter-stimulus interval (the time interval between each response and when the next cross appeared) ranged between 1 and 3 s and was randomized within these boundaries. Participants' mean reaction time across all trials is calculated and presented as a millisecond value which is used in subsequent analyses.

**(2) Cognitive memory recall** of the route as measured by a drawn map, post walk.

In order to capture participants' cognitive route recall, they were asked to draw sketch maps of each respective walk, completed immediately post walk. Following an unconstrained, route-based sketch mapping modality (29, 37), participants were given a blank page (no base map) with limited instructions, stating, “*Imagine you have a visitor who wants to undertake the route you just completed. Please draw the route you undertook so that the visitor could repeat this route*.” The cognitive sketch maps were reviewed by a member of the research team in order to assess five aspects of the maps: Usability, Accuracy, Network Quality, Waypoints, and Natural Features. These aspects of the map were selected based on prior literature establishing methods for assessing overall map quality (Usability, Accuracy, and Network Quality) as well as objective counts of specific map features (Waypoints and Natural Features) (38–40).

Nine participants completed sketch maps for each urban condition.

#### Physiological Measures

Real-time stress was captured during the walk using an android smart watch (Huawei Watch2) capturing heart rate and walking speed. We used an in-house built app to collect 100 HZ Photoplethysmogram (PPG), 60 Hz accelerometer, 1 HZ sound amplitude, and 1/60 HZ GPS data (41). The PPG signal used to estimate HRV was processed using bandpass filters to reduce motion artifacts. Heart rate is a bio indicator of the stress biologic system, and of SAM (sympathetic-adrenomedullary) system and HPA (hypothalamic-pituitary-adrenocortical) system activation, which work together to achieve allostasis, the body's ability to maintain stability through exposure to change and stressors. Heart rate variability captures the beat-to-beat interval variability of heart rate and is the most robust and consistent measure of physiological stress in real-time outdoor data capture (28).

#### Air Pollution and Noise Levels

Real time air-quality and noise measurements were collected during the walking sessions with handheld mobile devices. Air quality was measured by the Airbeam which detects particulate matter with a diameter equal to or smaller than 2.5 micron (PM_2.5_). Noise measurements were collected by an IK iRig lavalier microphone connected to a smartphone. Each device was held between 1 and 2 meters from the ground. The Airbeam is a low-cost air quality measurement device developed for community-based environmental assessment. While low-cost, California's Air Quality Sensor Performance Evaluation Center finds that Airbeam sensors “had good correlation with the [high performance sensor] from both the field (*R*^2^ ~ 0.65–0.70) and laboratory studies (*R*^2^ > 0.87)” (SCAQMD 2015). PM_2.5_ and noise (dB) readings were collected at least once per second as participants walked along their routes.

### Statistical Analyses

#### AirBeam Emissions and Noise Comparisons

An independent samples *t*-test was used to compare PM_2.5_ and dB readings between urban gray and urban green conditions for each day of the study. The *t*-test compares whether readings of particulate matter or noise levels in the two urban conditions are significantly different on a given day. In addition, effect sizes for each comparison were calculated using Cohen's d, which is the mean difference divided by the pooled standard deviation of readings for each urban condition.

#### SWEMWBS

Well-being scores, measured by SWEMWBS, were taken pre-walk on both days. A paired *t*-test was used to determine if SWEMWBS scores significantly differed between the two sessions. The paired *t*-test was used because measurement of SWEMWBS came prior to the walking sessions, so was not affected by the participants' route. Effect sizes were calculated using Cohen's d, calculated as described above.

#### UWIST

Change scores (post-walk score minus pre-walk score) were computed for each of the UWIST MACL components and analyzed using independent samples *t*-tests on each of the three outcome measures (Hedonic Tone, Stress and Arousal). This would determine any significant difference between the impact of the route on mood, as determined by the magnitude of the change scores. As with the SWEMWBS, effect sizes were calculated using Cohen's d, calculated as described above.

#### Simple Reaction Time

Two analyses were used to understand the SRT outputs. The first analysis was to understand if there were baseline differences between reaction times prior to the walking sessions on each day. A paired *t*-test was used to compare reaction times for each participant pre-walk on both their testing days (i.e., pre-urban gray vs. pre-urban green). The second analysis used change scores (post-walk reaction time – pre-walk reaction time) calculated for each walking session and used these in an independent samples *t*-test, using route as the grouping variable. As with previous *t*-test analyses, Cohen's d was calculated as described above.

#### Cognitive Maps

Cognitive maps elements were analyzed descriptively, assessing the mean number of cognitive map features (Usability, Accuracy, Network Quality, Waypoints, and Natural Features) drawn by participants in their sketch maps for urban gray and urban green walks.

#### HRV

Since data consisted of multiple physiological observations nested within individual participants, a multilevel random coefficient modeling approach with a random intercept for each participant was used. The models were fit using full information maximum likelihood estimation to study the effect of route types on HRV. The first model examines the relationship between HRV (RMSSD) and urban condition only. The second and third models examine the relationship between HRV and the interaction between urban condition and levels of either PM_2.5_ or dB.

## Results

### Demographics

Participant demographics from the study participants are presented in Table 2, below. One participant did not complete the 2nd walk, resulting in an overall sample of *n* = 11.

**Table 2:** Participant demographics.

**Age**	**Range**	**Mean**
	57–77 years	64.8 years
**Gender**	***N***	**Percentage**
Male	6	54.5%
Female	5	45.5%
**Ethnicity**		
White	8	72.2%
African-American	2	18.2%
Mixed race	1	9.1%
**Registered disability**		
Registered disabled	7	63.6%
Not registered disabled	4	34.4%
**Smoker status**		
Yes	3	27.3%
No	8	72.7%
**Income coping**		
Living very comfortably	1	9.1%
Living a little comfortably	1	9.1%
Living OK	2	18.2%
Living little difficultly	5	45.5%
Living very difficulty	2	18.2%
**Education level**		
None at all	1	9.1%
Primary school	3	27.3%
Secondary school	2	18.2%
Tertiary (college/university)	5	45.5%

### Environmental Measures: Noise and Air Pollution

#### Portable AirBeam Sensors

The AirBeam portable sensors supplied near-continuous (approx. every second) information on pollution and noise levels along the participants' walking routes. Table 3 describes PM_2.5_ and dB means and ranges for the urban gray and urban green walks the 2 days of the pilot. On Day 1, PM_2.5_ levels were higher during the urban green walk than during the urban gray walk. This was contrary to expectations but can be accounted for by local weather conditions and time of day. The urban green walk occurred later in the morning, and PM_2.5_ levels were building generally in Richmond that morning, even during the course of the urban gray walk. (See Supplementary Figures 3, 4, for a map of how PM_2.5_ levels varied during the course of the walks).

**Table 3:** Means and ranges for AirBeam measured PM_2.5_ (μg/m^3^) and dB levels during walks.

**Measure**	**Day**	**Environment**	**Mean**	**Min-max**	**Mean difference (Cohen's *d*)**
Particulate matter (PM_2.5_)	Day 1	Urban gray	15.85	1.94–35.28	−4.06*** (−0.848)
		Urban green	19.91	2.57–28.23	
	Day 2	Urban gray	9.88	0.86–25.21	1.28* (0.383)
		Urban green	8.60	1.03–13.49	
Noise level (dB)	Day 1	Urban gray	75.19	58.82–87.05	5.20*** (1.157)
		Urban green	69.99	59.39–84.91	
	Day 2	Urban gray	72.15	59.19–86.46	2.98*** (0.640)
		Urban green	69.17	58.88–81.59	

Mean difference between Urban Gray and Urban Green measures for given day and measure are significantly different at: *p < 0.05, **p < 0.01, ***p < 0.001.

Overall, Day 1 had higher PM_2.5_ levels than Day 2, which is reasonable considering the relatively warmer, less windy weather of the first day. Importantly, the mean PM_2.5_ concentrations (measured in μg/m^3^) were relatively low on both days. The World Health Organization recommends that PM_2.5_ concentrations should not exceed 25 μg/m^3^ over a 24-h mean, and these walks are lower than those levels during their short duration (42). While not included here, we also conducted mobile sampling of NO_2_ mixing ratios, where NO_2_ and PM_2.5_ are both associated with urban combustion pollution, the hour before and hour after participant walking times on Days 1 and 2. The mobile measurements captured general neighborhood-scale spatial patterns in vicinity of the walking routes. We observed that the spatial patterns collected using this repeated mobile sampling were not always consistent with those detected using the AirBeam. For example, on Day 1, mobile NO_2_ measurements were higher in the vicinity of the urban gray route than the urban green route, suggesting pollutant concentrations relevant to the scale of this study exhibited high spatiotemporal variability that required the use of the handheld monitoring devices.

Noise levels were consistently, significantly lower for the urban green walks relative to the urban gray walks, with moderate to large effect sizes. While ambient road noise is not directly regulated in Richmond, if from a single vehicle, the excursions above 86 dB in the urban gray walks would be violations of city regulations. Results indicate statistically significant differences between the two walk routes for both particulate matter (PM_2.5_) and noise level (dB).

### Psychological Outcomes

#### Subjective Well-Being

There was no significant difference between the SWEMWBS scores on the 2 days [*t*_(9)_ = 0.732, *p* = 0.483; *d* = 0.203] as revealed using a paired *t*-test to compare samples. The effect size shows only a small effect, further suggesting that the result is non-significant. The SWEMWBS scale is a sub-chronic measure of subjective well-being and shows that, over the duration of the study, subjective well-being was constant in our sample. Overall SWEMWBS scores on Day 1 were 27.4 (sd = 5.4) and on Day 2 were 25.9 (sd = 6.15).

#### Mood

**Figure 6:**
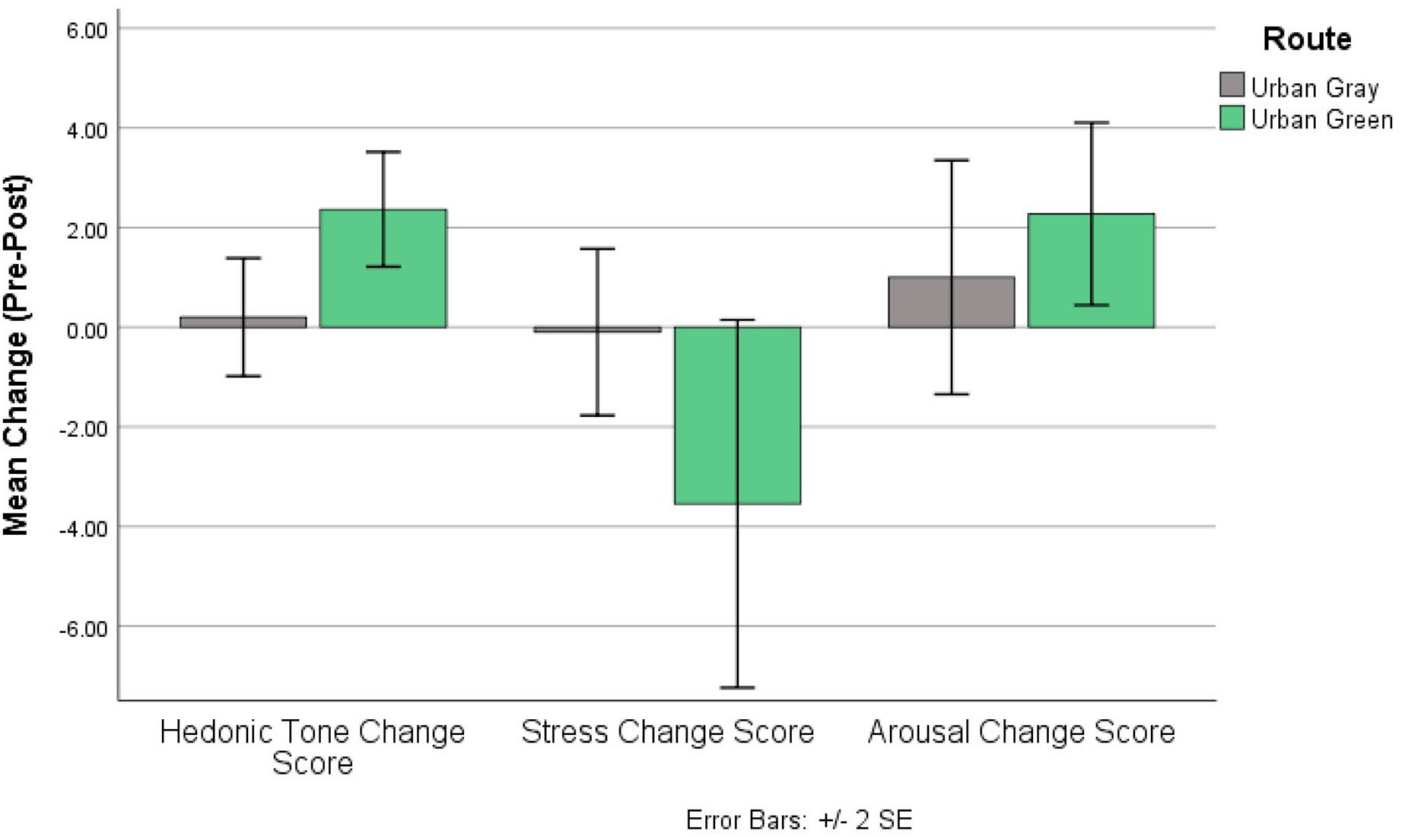
UWIST MACL change scores for each output; change scores generated from post-walk – pre-walk scores of each MACL output.

We examined the change scores between pre- and post-walk assessments of the UWIST MACL (mean scores provided in Table 4), allowing for reduced between subject variability. The results showed a significant effect of route type on hedonic tone (*t*_19_) = −2.62, *p* = 0.017; *d* = 1.14) but not on stress [*t*_19_ = 1.64, *p* = 0.117; *d* = 0.73] or arousal [*t*_19_ = −0.864, *p* = 0.399; *d* = 0.37]. Figure 6 shows the change scores; the only significant result is the increase in hedonic tone in the urban green condition when compared to the urban gray condition. However, Figure 6 also shows the hypothesized direction of change for each non-significant condition; stress decreases post-walk at a larger rate than the urban gray (the standard error bars suggest, however, a varied response between participants) and arousal increases in the urban green relative to the urban gray condition. The effect scores show a large effect of route on hedonic tone (1.14), supporting the significance of this result. We also see a medium effect size of route on stress (0.73), suggesting, with appropriately powered participant numbers, there may be an overarching effect. The effect size of route on arousal is small-to-medium (0.37), suggesting there may not be an effect of route on arousal, supported by the non-significant result.

**Table 4:** Psychological outcomes for subjective wellbeing outcomes (standard deviations in parentheses).

**SWEMWBS**	**Day 1**	**Day 2**
	27.45 (5.47)	25.9 (6.15)
**UWIST MACL**	**Pre-walk**	**Post-walk**
Hedonic tone: urban gray	26.6 (3.5)	26.8 (3.85)
Hedonic tone: urban green	24.91 (4.85)	27.27 (4.84)
Stress: urban gray	14.7 (4.81)	14.6 (4.74)
Stress: urban green	16.55 (5.36)	13 (4.05)
Arousal: urban gray	23.9 (3.96)	24.9 (3.96)
Arousal urban green	23 (4.27)	25.27 (3.69)
**Simple reaction time (ms)**		
Urban gray	436.36 (155.88)	459.1 (122.01)
Urban green	427.11 (95.85)	412.64 (134.3)

*SWEMWBS scores range between 7 and 35; all three MACL outcome scores range between 8 and 32*.

#### Short Reaction Time Test

**Figure 7:**
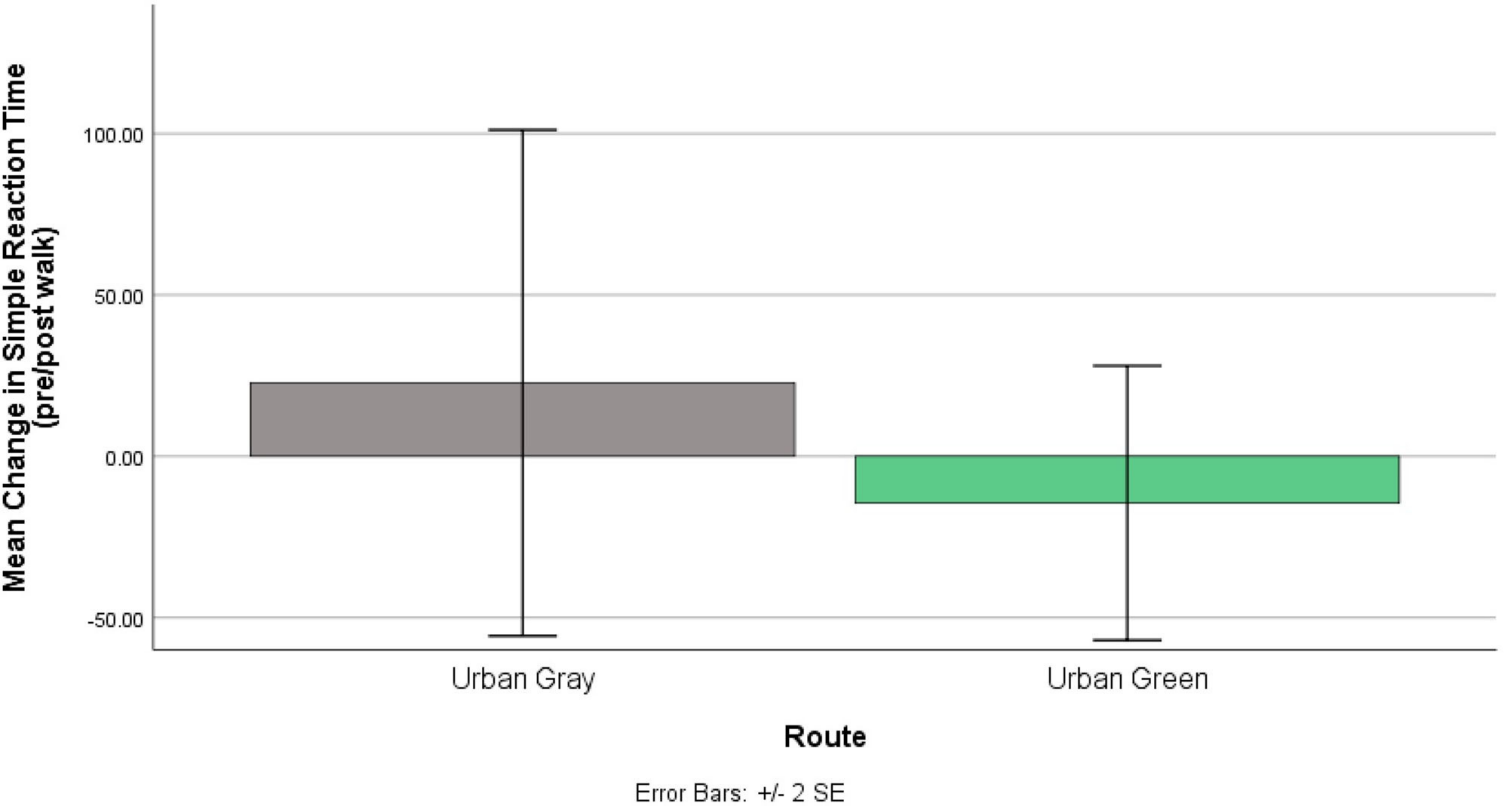
Changes to Short Cognitive Reaction time (ms) pre and post walks. Urban gray shows an increased reaction time while urban green shows decreased reaction time both post-walk.

Table 4 shows the mean reaction times (ms) pre- and post-walk for the urban gray vs. urban green walk. Participants were counterbalanced between the two conditions to ensure no order effects would be present in the results. Initially, we wanted to check if there were statistically significant differences between the pre-walk conditions (irrespective of the walk). We found no significant difference between baseline (pre-walk) SRT reaction times on each study day [*t*_(9)_ = 0.32, *p* = 0.756; *d* = 0.11], suggesting that any difference between pre- and post-walk SRT score is likely due to condition effects rather than a skewed baseline, supported further by the low effect size. An independent t-test was then used to assess the change from baseline score (post-walk reaction time – pre-walk reaction) and showed no significant effect of route type on performance [*t*_(19)_ = 0.854, *p* = 0.403; *d* = 0.37). Figure 7, however, shows that participants' reaction times improved (i.e., got faster) post-urban green walks compared to reaction times getting slower post-urban gray walks, but the effect size shows that the strength of this effect is small (0.37).

#### Cognitive Maps

Table 5 presents the cognitive mapping results for the respondents. Overall, most respondents took similar approaches to cognitive mapping, and average usability, accuracy, network quality, and waypoint count were similar between urban gray and urban green maps. Natural features were only used in two of nine urban green maps, but waypoints (landmarks between origin and destination) were included more frequently in the sketch maps of the urban green route.

**Table 5:** Cognitive map assessments by participant.

	**Environment type**	**Mean score/count**
Usability	Urban gray	2.8
	Urban green	2.7
Accuracy	Urban gray	3.4
	Urban green	3.3
Network quality	Urban Gray	3.1
	Urban green	3.2
Waypoints	Urban gray	0.7
	Urban green	1.6
Natural features	Urban gray	0.0
	Urban green	0.2

*Usability - Could the map objectively be used to give directions to another person? (1 to 5)*.

*Accuracy - Does the map conform to the actual geography of the route? North does not need to be up (1 to 5)*.

*Network - How refined is the network? A highly refined network would label streets, show routing, and use additional streets for context (1 to 5)*.

*Waypoints - How many discernable waypoints (landmarks between origin and destination) does the map include? (Count)*.

*Nature - How many waypoints representative of nature? (Count)*.

### Physiological Data

**Figure 8:**
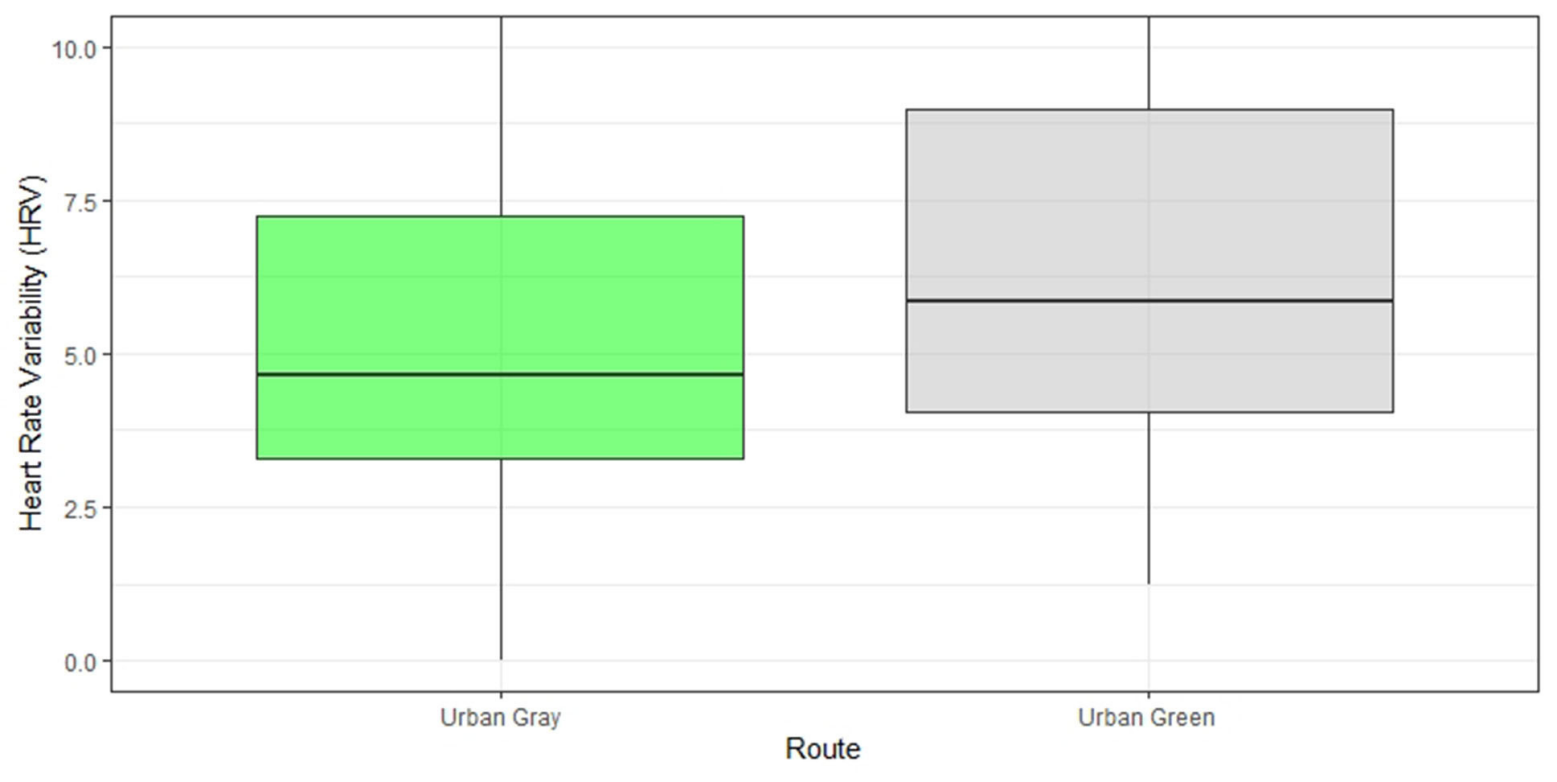
Heart rate variability difference post walk in the “green” vs. “gray” condition. HRV is represented by the Root Mean Square of the Successive Differences (RMSSD). *Note: a lower heart rate variability indicates higher cardiac activation and higher stress*.

Figure 8 shows the difference between the two conditions for HRV. To characterize HRV, we computed the Root Mean Square of the Successive Differences (RMSSD), a well-validated and most accurate measure of Autonomic Nervous System activity (43). HRV is computed and compared across the two conditions in a linear mixed effect model (see Table 6, Model 1). The multilevel model revealed a significant effect of walking in an urban gray condition on HRV (*b* = −2.16, *p* < 0.001). Since lower HRV is associated with elevated stress, our finding indicates that the urban gray condition increased stress levels when compared to the urban green condition.

**Table 6:** Associations Between Environmental Conditions and Physiological Responses by Urban Setting (Green vs. Gray).

**Linear mixed effects models: dependent variable HRV (RMSSD)**			
	**Model 1**	**Model 2**	**Model 3**
**Fixed effects**	**Coefficients**		
Urban gray (vs. Urban green)	−2.1689***	16.8136***	−2.0007***
dB		0.0929*	
Urban gray x dB		−0.2609***	
PM_2.5_			−0.1421***
Urban gray x PM_2.5_			0.03904
Intercept	9.8012***	1.70934	
**Random effects**	**Standard deviations**		
Intercept	1.5690	2.4972	2.3919
Residuals	8.3229	7.4065	7.3910
**Model diagnostics**			
AIC	9,293.247	36,207.37	36,184.46
BIC	9,313.959	36,246.8	36,223.89
*N*		5,284	5,284

Coefficients are significantly different at: *p < 0.05, **p < 0.01, ***p < 0.001.

*Model 1: Interaction between urban conditions and HRV; Model 2: Interaction effect of dB and urban conditions on HRV; Model 3; Interaction effect of PM_2.5_ and urban conditions on HRV*.

### Synthesis of Data

**Figure 9:**
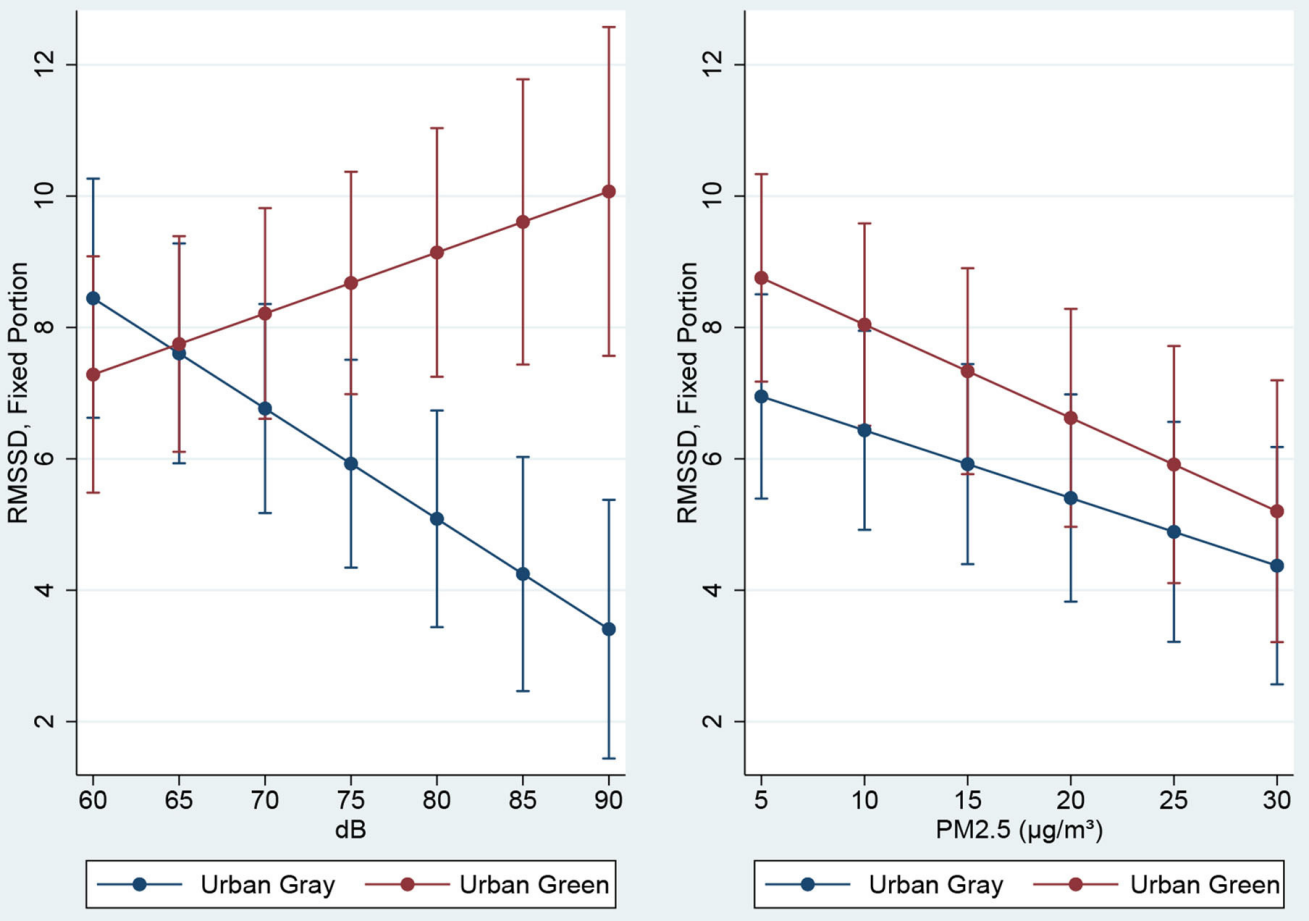
Heart rate variability (RMSSD) by “green” vs. “gray” condition over observed values of dB and PM_2.5_ *Note: a lower heart rate variability indicates higher cardiac activation and higher stress*.

We examined relationships between the environmental and the physiological data collected while participants were walking the urban green and urban gray routes. Table 6, Models 2 and 3, show results of linear mixed effects models examining the effect of environmental measures (PM_2.5_ or dB) and their interactions with urban conditions. To aid interpretation of the interaction terms, Figure 9 illustrates the fixed effects relationships among urban conditions, environmental measure, and heart rate variability measured as RMSSD, at the ranges for the environmental values measured during the study. Across measures of dB, RMSSD is similar at low noise levels in both the urban green and urban gray conditions, but as dB increases, RMSSD diverges with lower readings (more stress) in the urban gray but relatively even stress levels (taking confidence intervals into account) in the urban green condition. For PM_2.5_, RMSSD decreases (more stress) as particulate matter increases at similar rates in both urban conditions. The difference between RMSSD in the two urban conditions is significant in the model (Table 6, Model 3).

Given a significant relationship between noise and stress, we ran further data analyses to explore relationships between noise (dB) and cognitive functioning [i.e., simple reaction times (SRT)]. Results showed no significant effect of noise on SRT.

## Discussion

Based on prior research evidence, we posited that older people, on a low income, will benefit from walking in local neighborhoods that include urban street trees, and other urban natural features such as domestic gardens and nearby parks, as measured by indicators of subjective well-being, stress and cognition (Hypothesis 1).

First, for subjective well-being, we found a statistically significant difference between the two walk routes (i.e., urban gray *vs*. urban green) on hedonic tone, which increased more from walking in the urban green route; this is consistent with research showing mood benefits from walking in green *vs*. gray conditions (24, 44). Findings on indicators of arousal (energetic vigor) and perceived stress, whilst not significant, indicate positive outcomes aligning with our hypotheses for the green walk as compared to the gray route. Higher hedonic tone in the green condition is an important outcome, representing an increased capacity to experience pleasure, and a reduced risk of experiencing anhedonia, one of the symptoms of depression (45). Older people are at greater risk of depression owing to increases in adverse life events (e.g., loss of a spouse or close friend), social isolation and financial stressors (46), therefore increasing hedonic capacity in older people via access to walkable, green urban conditions may have important implications for mental health.

Second, on physiological stress outcomes, we found significant differences between walk routes for heart rate and heart rate variability (HRV) with lower stress activation from walking in the urban green route. Our stress response is a complex process that involves two interrelated biologic systems: the sympathetic nervous system that triggers the “flight or fight” response (and provides the body with the energy to take action), and the parasympathetic nervous system that acts as a break (promoting “rest and digest” and calming the body down). Our results for HRV and heart rate suggest walking in green space activates the parasympathetic system and induces a calming effect. This finding is consistent with earlier research in non-laboratory settings showing a positive effect on stress regulation from exposure to green space outdoors (28) as well as increases in alpha brain activity associated with increased relaxation (25). HRV is one of the most consistently and reliable measures of stress physiology in this field of research and, albeit in a small sample size, our results show promising findings for real-time stress monitoring in older people.

The sympathetic and parasympathetic nervous systems work together to achieve allostasis, or the body's ability to maintain stability through change. Allostasis is important for maintaining good stress resilience. Reduced allostatic load (AL) has been associated with higher levels of green space in the neighborhood environment (47). Among older people, AL has been associated with cardiovascular disease, physical decline, cognitive function and depression (48–50). In addition, greater amounts of green space are associated with increased physical activity, and less stress (as measured by self-report) in older people. Alleviation of chronic stress for older people is therefore one important pathway by which to improve overall health. Easy access to walkable residential neighborhoods with green space is therefore one important public health intervention that can support healthy aging.

Third, on cognitive health outcome measures, short reaction time (SRT) results indicate faster reaction times post-urban green walk, albeit not significant in this small sample. This has implications for maintaining mental alertness whilst walking, reducing the risk of trips and falls amongst older people outdoors. The SRT task is a measure of speed of attention and processing, so further investigation is needed to understand if green spaces can reliably improve attentional capacity in older populations. It also supports previous behavioral research that shows improved directed-attention performance following exposure to walks in nature (51, 52).

On indicators of spatial memory, participants' practical ability to draw usable, accurate route sketch maps did not vary between the two urban conditions. However, waypoints were included at double the rate in the urban green setting compared to the urban gray setting, though with the small sample size (*N* = 11 complete sketch map sets), this mean difference was only significant at the *p* < 0.1 level. The fact that the urban green route did prompt a more detailed recollection of the features of the route, compared to the urban gray route, is worth further consideration. It is possible that the architectural qualities of the urban green route, which proceeded through a historic area, were more memorable than those of the urban gray route on busy Broad Street. Alternatively, the relative calm of the urban green route may have enabled greater attention to surroundings, which could improve recall of waypoints. The literature on variations in cognitive recall of the environment under conditions of stress is limited, and further research in this area could help explain the process by which features of the environment do or do not accrue meaning and value for local residents.

Fourth, we posited that lower levels of air and noise pollution will result in improvements across the mental health outcome measures (Hypothesis 2). We found significant interaction effects between levels of air / noise pollution, urban conditions and stress activation. First, stress levels increased with increasing noise levels (dB) in the urban gray condition; by comparison, stress activation decreased in the urban green condition as dB increased (Figure 9). But the effect of noise (dB) on stress activation was stronger from walking in a busy, trafficked urban walk. Chronic noise keeps the body's stress response constantly activated contributing to increased risk of heart disease and mood disturbances (reflected in our finding on hedonic tone above). It appears that urban greenery may be acting as a buffer to stress activation from increasing dB; it's possible we are more tolerant to increasing noise in urban green conditions, a proposition warranting further research. We also found significant effects of air pollution (PM_2.5_) and urban conditions on stress activation; in both conditions stress increased as PM_2.5_ increased, but with no potential buffering effect of green space, although stress activation was greater in the trafficked urban walk (Figure 9). Given that other studies have identified associations between mental health (i.e., self-reported anxiety, depression and stress) and heart rate dynamics (i.e., HRV) and exposure to traffic and air pollutants in healthy adults (53, 54), in a larger sample we might expect to see a stronger effect of urban conditions and air pollution on stress activation. It is also possible that a longer exposure time to air pollution may be required to detect differences. A 2-hour walk, for example, on a traffic-polluted street has shown adverse effects on older people's cardio-respiratory health (55). In summary, we found significant interaction effects between levels of noise/air pollution, urban conditions and stress activation, with a stronger effect for noise in busy trafficked conditions. Our study therefore warrants further examination of air-pollution-traffic-mental health associations using mobile health and environmental sensors.

This pilot study seeks to understand relationships between environmental and physiological data, and integrates near-continuous, precise data streams on measures including noise, particulate matter pollution, and heart rate variability. Our protocols utilize both time and place records to match data streams. We established a process to then observe co-variation across time and place, both at the level of individuals and across shared experiences. While our pilot sample was small, these processes were effective and can be applied to larger samples in future research. Increasing sample size will increase power and may lead to statistical significance.

In addition, we also wanted to test the viability of recruiting and implementing a complex study protocol in senior people on lower incomes. There are many challenges to using mobile human health and environmental data sensors. The technology is relatively new, largely tested in younger (student) populations, and its application requires testing in a wider participant demographic. We anticipated it may be challenging for senior people to wear a mobile sensor and comply with stringent criteria about eating, drinking and talking during the experiment, given that our participants – whilst living independently – were also experiencing financial stressors from retirement, and some health challenges (7 participants with a registered disability). A total of 11 participants (from a sample of 12 recruited) were able to comply with our study protocol across two walk days with an intervening period of 1 day between walk days. The older people in our experiment did find some pre- and post-walk tasks difficult (e.g., the short reaction time task was substituted for a complex reaction time task, tested in a pilot, which our participants found mentally challenging).

We did not explore heat stress, but the alleviation of heat stress by tree canopy cover has a significant role to play in aiding mobility for older people during hot summer periods. Urban greenery (e.g., street trees, parks, and front gardens) may reduce surface temperatures to which older populations are more sensitive, highlighting the importance of green infrastructure for “older neighborhoods” associated with particular older demographic groups. This is the focus of a follow-on study.

## Limitations

Our study captured acute stress, and immediate physiologic changes over a short-term period using heart rate measures. Capturing chronic stress requires the repeated capture of diurnal cortisol over time, or measures of allostatic load (indicators of immune, neuroendocrine, and metabolic function) typically captured in saliva.

Our participants self-selected to participate in the study, therefore, bias may have occurred due to non-random selection. We cannot demonstrate any causal mechanisms between outdoor exposure, stress and health. Using RCTs (randomized control trials) – along with bigger samples – is required to increase the generalizability of results. However, given the challenges of combining multiple heterogeneous sensor data streams (e.g., HRV with air pollution) which have different scales, sampling rates and missing data patterns, and due to the complexity of integrating real-time environmental and physiological data sets (with billions of milliseconds of data) with subjective data, the research field will likely grow slowly with small samples to establish robust protocols for both experimental design and data processing/integration approaches. Sampling sizes tend to be small, however, our study, employing a cross-over design and the capture of pre and post data in two dichotomous environments, improves on the experimental work characteristic of the field to date [see (28)].

Given that associations between urban green space and mental health outcomes in older people are significantly modified by race, age, gender, social support, physical function, and socioeconomic status (53, 56) it is important that future research sufficiently power participant sample sizes to explore differences by socio-demographic characteristics. Other variables of interest to future studies exploring mental health-built environment associations in older people include aesthetics (including littering/vandalism/order as well as greenery) which are associated with increased physical activity in older people (57), and walk pace, which is associated with increased life longevity (58).

Our study has taken a closer step toward measuring ways that the local nearby environment relates to human health in older people, which will improve our ability to identify more nuanced relationships between environmental exposure, stress and cognitive health outcomes. We have begun to set out a process by which a complex matrix of physiologic and environmental data can be integrated. Further development of methodological approaches (e.g., use of time-activity monitoring across space and time, developing data fusion techniques to analyze the multimodal data) could help characterize the complex matrix of social and physical circumstances, both indoors and outdoors, that contribute to or mitigate stress. Additionally, given the potential benefits of exposure to natural spaces on stress and cognitive functioning, interventions paired with research are needed most in those neighborhoods that lack close proximity to such space.

## Conclusion

Our study increases understanding of how walking in the immediate neighborhood environment affects stress and other health indicators in older people. In a vulnerable aging population, we successfully established a protocol for measuring ambulatory heart rate using mobile technologies and correlating them with urban analytical data captured in real-time outdoors. This is the first time, to our knowledge, that the impact of urban greening on real-time stress responses in older populations has been examined using these methods.

As the life longevity and the number of older persons increases globally, a central question is, what changes in the built environment can improve quality of life and sustain health for an aging population? Albeit in a small sample, our study suggests that publicly accessible urban green space offers promising opportunities for supporting mental health in older people. Whilst further research is needed, findings can inform urban planning for health, and help inform global initiatives such as the World Health Organization's ‘Age Friendly Cities' program.

## Data Availability Statement

The raw data supporting the conclusions of this article will be made available by the authors, without undue reservation.

## Ethics Statement

The studies involving human participants were reviewed and approved by University of Virginia IRB Health Sciences. The patients/participants provided their written informed consent to participate in this study.

## Author Contributions

JR conceptualized the study design, oversaw participant recruitment, data collection, data analyses and interpretation of findings, and led the writing of the manuscript with input from co-authors. AM led the cognitive mapping exercise, methods for air/pollution data collection, and carried out the data synthesis with physiological data. CN assisted with the study protocol and led the subjective data analyses. LB and MB led the methods and analyses for the real-time stress data capture. SL led the land use data analyses and assisted with the preparation of the manuscript. All authors assisted with data collection in the field.

## Conflict of Interest

The authors declare that the research was conducted in the absence of any commercial or financial relationships that could be construed as a potential conflict of interest.

## References

[1] Rovio S Kåreholt I Helkala E Viitanen M Winblad B Tuomilehto J. Leisure-time physical activity at midlife and the risk of dementia and Alzheimer's disease. Lancet Neurol. (2005) 4:705–11. 10.1016/S1474-4422(05)70198-8 16239176

[2] Hörder H Johansson L Guo X Grimby G Kern S Östling S. Midlife cardiovascular fitness and dementia. Neurology. (2018) 90:e1298. 10.1212/WNL.0000000000005290 29540588 PMC5894933

[3] Erickson KI Voss MW Prakash RS Basak C Szabo A Chaddock L. Exercise training increases size of hippocampus and improves memory. Proc Natl Acad Sci USA. (2011) 108:3017–22. 10.1073/pnas.1015950108 21282661 PMC3041121

[4] Won J Alfini AJ Weiss LR Michelson CS Callow DD Ranadive SM. Semantic memory activation after acute exercise in healthy older adults. J Int Neuropsychol Soc. (2019) 25:557–68. 10.1017/S1355617719000171 31018875

[5] Smith JC Nielson KA Antuono P Lyons J Ryan Hanson J Butts AM. Semantic memory functional MRI and cognitive function after exercise intervention in mild cognitive impairment. J Alzheimer Dis JAD. (2013) 37:197–215. 10.3233/JAD-130467 23803298 PMC4643948

[6] Macpherson H Teo W Schneider LA Smith AE. A life-long approach to physical activity for brain health. Front Aging Neurosci. (2017) 9:147. 10.3389/fnagi.2017.00147 28588474 PMC5440589

[7] van den Berg PEW Kemperman ADM de Kleijn B Borgers AWJ Ageing and loneliness: the role of mobility and the built environment. Travel Behav Soc. (2016) 5:48–55. 10.1016/j.tbs.2015.03.001

[8] CDC Physical Activity Guidelines for Americans, 2nd Edition. U.S. Department of Health and Human Services (2018).

[9] Zenko Z Willis EA White DA. Proportion of Adults Meeting the 2018. Physical activity guidelines for Americans according to accelerometers. Front Pub Health. (2019) 7:135. 10.3389/fpubh.2019.00135 31231627 PMC6566056

[10] Livingston G Sommerlad A Orgeta V Costafreda SG Huntley J Ames D. Dementia prevention, intervention, and care. Lancet. (2017) 390:2673–734. 10.1016/S0140-6736(17)31363-6 28735855

[11] Peters R Ee N Peters J Booth A Mudway I Anstey KJ. Air pollution and dementia: a systematic review. J Alzheimer Dis. (2019) 70:S145–63. 10.3233/JAD-180631 30775976 PMC6700631

[12] Carey IM Anderson HR Atkinson RW Beevers SD Cook DG David Strachan P. Are noise and air pollution related to the incidence of dementia? A cohort study in London, England. BMJ Open. (2018) 8:e022404. 10.1136/bmjopen-2018-022404 30206085 PMC6144407

[13] Choi W He M Barbesant V Kozawa KH Mara S Winer AM. Prevalence of wide area impacts downwind of freeways under pre-sunrise stable atmospheric conditions. Atmospheric Environ. (2012) 62:318–27. 10.1016/j.atmosenv.2012.07.08425379010

[14] Karner AA Eisinger DS Niemeier DA. Near-roadway air quality: synthesizing the findings from real-world data. Environ Sci Technol. (2010) 44:5334–44. 10.1021/es100008x 20560612

[15] Adar SD Kaufman JD. Cardiovascular disease and air pollutants: evaluating and improving epidemiological data implicating traffic exposure. Inhalation Toxicol. (2007) 19(Suppl. 1):135–49. 10.1080/08958370701496012 17886061

[16] Lipfert FW Wyzga RE. On exposure and response relationships for health effects associated with exposure to vehicular traffic. J Exposure Sci Environ Epidemiol. (2008) 18:588–99. 10.1038/jes.2008.4 18322450

[17] Basner M Babisch W Davis A Brink M Clark C Janssen S. Auditory and non-auditory effects of noise on health. Lancet. (2014) 383:1325–32. 10.1016/S0140-6736(13)61613-X 24183105 PMC3988259

[18] McAlexander TP Gershon RRM Neitzel RL. Street-level noise in an urban setting: assessment and contribution to personal exposure. Environ Health. (2015) 14:18. 10.1186/s12940-015-0006-y 25888945 PMC4350859

[19] City of Richmond Excessive Noise Ordinance. (2011). Available online at: http://media.rvanews.com/wp-content/uploads/2011/09/noise-ordinance.pdf (accessed June 1, 2020).

[20] Allen J Balfour R Natural Solutions for Tackling Health Inequalities. London: UCL Institute of Health Equity; Natural England (2014).

[21] Dennis M Scaletta KL James P. Evaluating urban environmental and ecological landscape characteristics as a function of land-sharing-sparing, urbanity and scale. PLoS ONE. (2019) 14:e0215796. 10.1371/journal.pone.0215796 31344035 PMC6657829

[22] Takano T Nakamura K Watanabe M. Urban residential environments and senior citizens' longevity in megacity areas: the importance of walkable green spaces. J Epidemiol Commun Health. (2002) 56:913–8. 10.1136/jech.56.12.913 12461111 PMC1756988

[23] Sulander T Karvinen E Holopainen M. Urban Green space visits and mortality among older adults. Epidemiology. (2016). 27:e34–5 10.1097/EDE.0000000000000511 27327021

[24] Roe J Aspinall P. The restorative benefits of walking in urban and rural settings in adults with good and poor mental health. Health Place. (2011) 17:103–13. 10.1016/j.healthplace.2010.09.003 21094074

[25] Neale C Aspinall P Roe J Tilley S Mavros P Cinderby S The impact of walking in different urban environments on brain activity in older people. Cities Health. (2019) 4:1 10.1080/23748834.2019.1619893

[26] Hartig T Mitchell R de Vries S Frumkin H. Nature and health. Ann Rev Pub Health. (2014) 35:207–28. 10.1146/annurev-publhealth-032013-182443 24387090

[27] Wolch JR Byrne J Newell JP urban green space, public health, and environmental justice: the challenge of making cities just green enough. Landscape Urban Plan. (2014) 125:234–44. 10.1016/j.landurbplan.2014.01.017

[28] Kondo MC Fluehr JM McKeon T Branas CC. Urban green space and its impact on human health. Int J Environ Res Pub Health. (2018) 15:445. 10.3390/ijerph15030445 29510520 PMC5876990

[29] Mondschein A Blumenberg E Taylor B Accessibility and cognition: the effect of transport mode on spatial knowledge. Urban Stud. (2010) 47:845–66. 10.1177/0042098009351186

[30] Loukaitou-Sideris A Gilbert L Shades of duality: perceptions and images of downtown workers in Los Angeles. J Archit Plan Res. (2000) 17:16–33. 10.1080/02513625.2000.10556729

[31] Iaria G Palermo L Committeri G Barton JJS. Age Differences in the formation and use of cognitive maps. Behav Brain Res. (2009) 196:187–91. 10.1016/j.bbr.2008.08.040 18817815

[32] Aspinall P Mavros P Coyne R Roe J. The Urban Brain: analysing outdoor physical activity with mobile EEG. Br J Sports Med. (2015) 49:272–6. 10.1136/bjsports-2012-091877 23467965

[33] Neale C Aspinall P Roe J Tilley S Mavros P Cinderby S The aging urban brain: analyzing outdoor physical activity using the emotiv affectiv suite in older people. J Urban Health. (2017) 94:869–80. 10.1007/s11524-017-0191-9 28895027 PMC5722728

[34] Matthews G Jones DM Chamberlain AG Refining the measurement of mood: the UWIST mood adjective checklist. Br J Psychol. (1990) 81:17–42. 10.1111/j.2044-8295.1990.tb02343.x

[35] Schultheiss OC Brunstein JC Goal imagery: bridging the gap between implicit motives and explicit goals. J Personal. (1999) 67:1–38. 10.1111/1467-6494.00046

[36] Deary IJ Liewald D Nissan J. A free, easy-to-use, computer-based simple and four-choice reaction time programme: the deary-liewald reaction time task. Behav Res Methods. (2011) 43:258–68. 10.3758/s13428-010-0024-1 21287123

[37] Chorus CG Timmermans HJP Determinants of stated and revealed mental map quality: an empirical study. J Urban Design. (2010) 15:211–26. 10.1080/13574801003638095

[38] Cohen R Baldwin LM Sherman RC Cognitive maps of a naturalistic setting. Child Dev. (1978) 49:1216–8. 10.2307/1128763

[39] Rovine MJ Weisman GD Sketch-map variables as predictors of way-finding performance. J Environ Psychol. (1989) 9:217–32. 10.1016/S0272-4944(89)80036-2

[40] Bjerva T Sigurjónsson T Wayfinding by means of maps in real-world settings: a critical review. The J Navigat. (2017) 70:263–75. 10.1017/S0373463316000643

[41] Boukhechba M Barnes L SWear: Sensing Using WEARables. Generalized Human Crowdsensing on Smartwatches. In. (2020). 10.1007/978-3-030-51828-8_67

[42] World Health Organization Ambient (Outdoor) Air Pollution. (2018). Available online at: https://www.who.int/news-room/fact-sheets/detail/ambient-(outdoor)-air-quality-and-health (accessed June 1, 2020).

[43] Berntson GG Lozano DL Chen Y. Filter Properties of root mean square successive difference (RMSSD) for heart rate. Psychophysiology. (2005) 42:246–52. 10.1111/j.1469-8986.2005.00277.x 15787862

[44] Tilley S Neale C Patuano A Cinderby S. Older people's experiences of mobility and mood in an urban environment: a mixed methods approach using electroencephalography (EEG) and interviews. Int J Environ Res Pub Health. (2017) 14:151. 10.3390/ijerph14020151 28165409 PMC5334705

[45] Sternat T Katzman MA. Neurobiology of hedonic tone: the relationship between treatment-resistant depression, attention-deficit hyperactivity disorder, and substance abuse. Neuropsychiatr Disease Treatment. (2016) 12:2149–64. 10.2147/NDT.S111818 27601909 PMC5003599

[46] Pocklington C. Depression in older adults. BJMP. (2017) 10:7.26890937

[47] Egorov AI Griffin SM Converse RR Styles JN Sams EA Wilson A Vegetated land cover near residence is associated with reduced allostatic load and improved biomarkers of neuroendocrine, metabolic and immune functions. Environ Res. (2017) 158:508–21. 10.1016/j.envres.2017.07.009 28709033 PMC5941947

[48] Juster RP McEwen BS Lupien SJ. Allostatic load biomarkers of chronic stress and impact on health and cognition. Neurosci Biobehav Rev. (2010) 35:2–16. 10.1016/j.neubiorev.2009.10.002 19822172

[49] Seeman TE McEwen BS Rowe JW Singer BH. Allostatic load as a marker of cumulative biological risk: macarthur studies of successful aging. Proc Natl Acad Sci USA. (2001) 98:4770–5. 10.1073/pnas.081072698 11287659 PMC31909

[50] Seeman TE Singer BH Rowe JW Horwitz RI McEwen BS. Price of adaptation–allostatic load and its health consequences. MacArthur studies of successful aging. Arch Intern Med. (1997) 157:2259–68. 10.1001/archinte.157.19.2259 9343003

[51] Berman MG Jonides J Kaplan S. The cognitive benefits of interacting with nature. Psychol Sci. (2008) 19:1207–12. 10.1111/j.1467-9280.2008.02225.x 19121124

[52] Berman MG Kross E Krpan KM Askren MK Burson A Deldin PJ. Interacting with nature improves cognition and affect for individuals with depression. J Affect Disord. (2012) 140:300–5. 10.1016/j.jad.2012.03.012 22464936 PMC3393816

[53] Pun VC Manjourides J Suh HH. Close proximity to roadway and urbanicity associated with mental ill-health in older adults. Sci Total Environ. (2019) 658:854–60. 10.1016/j.scitotenv.2018.12.221 30583181 PMC7004241

[54] Meier-Girard D Delgado-Eckert E Schaffner E Schindler C Künzli N Adam M. Association of long-term exposure to traffic-related PM10 with heart rate variability and heart rate dynamics in healthy subjects. Environ Int. (2019) 125:107–16. 10.1016/j.envint.2019.01.031 30716571

[55] Sinharay R Gong J Barratt B Ohman-Strickland P Ernst S Kelly FJ Respiratory and cardiovascular responses to walking down a traffic-polluted road compared with walking in a traffic-free area in participants aged 60 years and older with chronic lung or heart disease and age-matched healthy controls: a randomised, crossover study. Lancet. (2018) 391:339–49. 10.1016/S0140-6736(17)32643-0 29221643 PMC5803182

[56] Pun VC Manjourides J Suh HH. Association of neighborhood greenness with self-perceived stress, depression and anxiety symptoms in older U.S adults. Environ Health. (2018) 17:39. 10.1186/s12940-018-0381-2 29661194 PMC5902952

[57] Rachele JN Sugiyama T Davies S Loh VHY Turrell G Carver A. Neighbourhood built environment and physical function among mid-to-older aged adults: a systematic review. Health Place. (2019) 58:102137. 10.1016/j.healthplace.2019.05.015 31176106

[58] Zaccardi F Davies MJ Khunti K Yates T. Comparative relevance of physical fitness and adiposity on life expectancy: a UK biobank observational study. Mayo Clin Proc. (2019) 94:985–94. 10.1016/j.mayocp.2018.10.029 31079962

