# Supplementary material for: The Urban Built Environment, Walking and Mental Health Outcomes Among Older Adults: A Pilot Study
Source: Front Public Health. 2020 Sep 23;8:575946. doi: 10.3389/fpubh.2020.575946 (PMC7538636; doi:10.3389/fpubh.2020.575946)

Supplementary Material

Supplementary Figure 1: Urban Green Walk Landcover

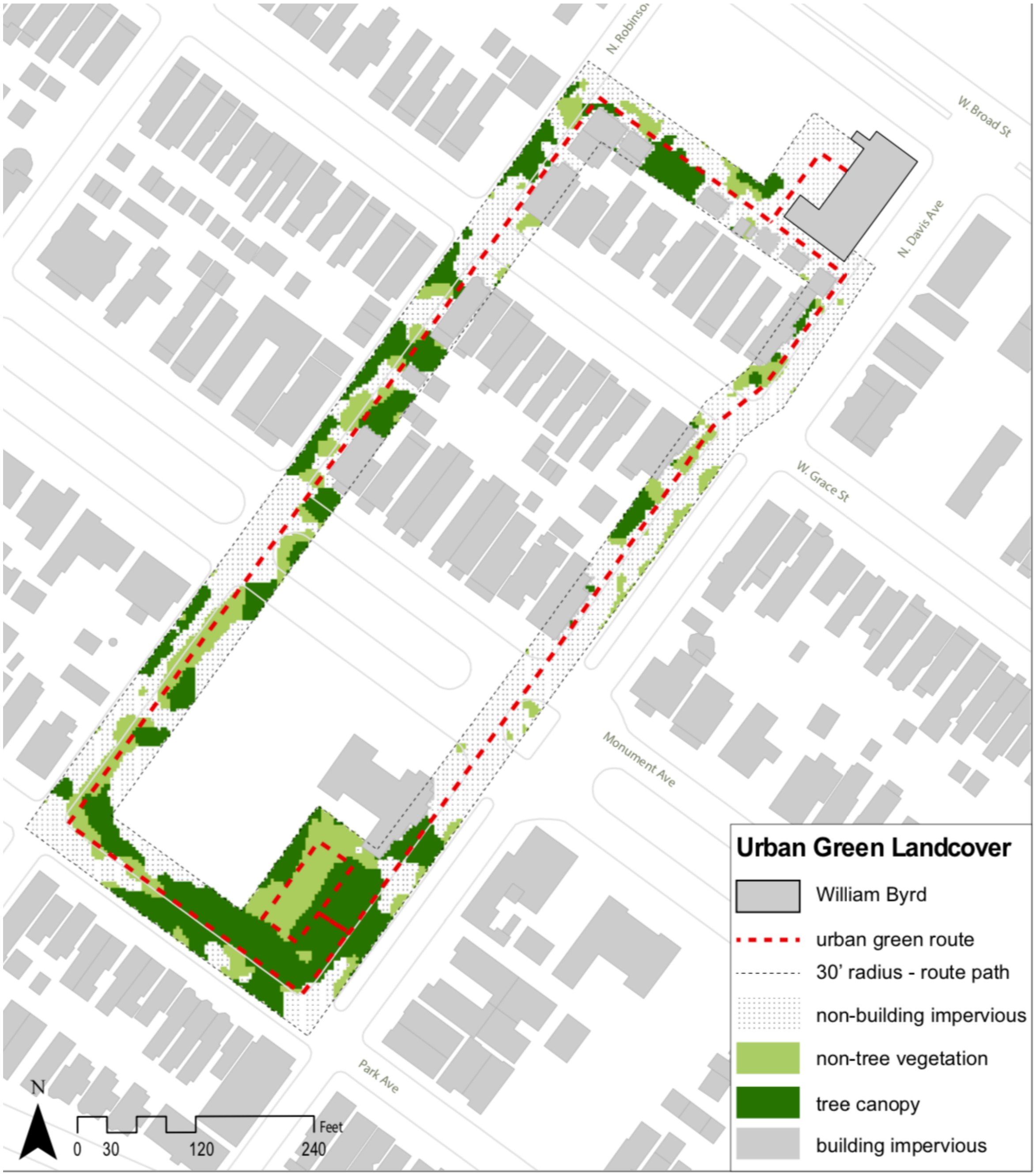

Supplementary Figure 2: Urban Gray Walk Landcover

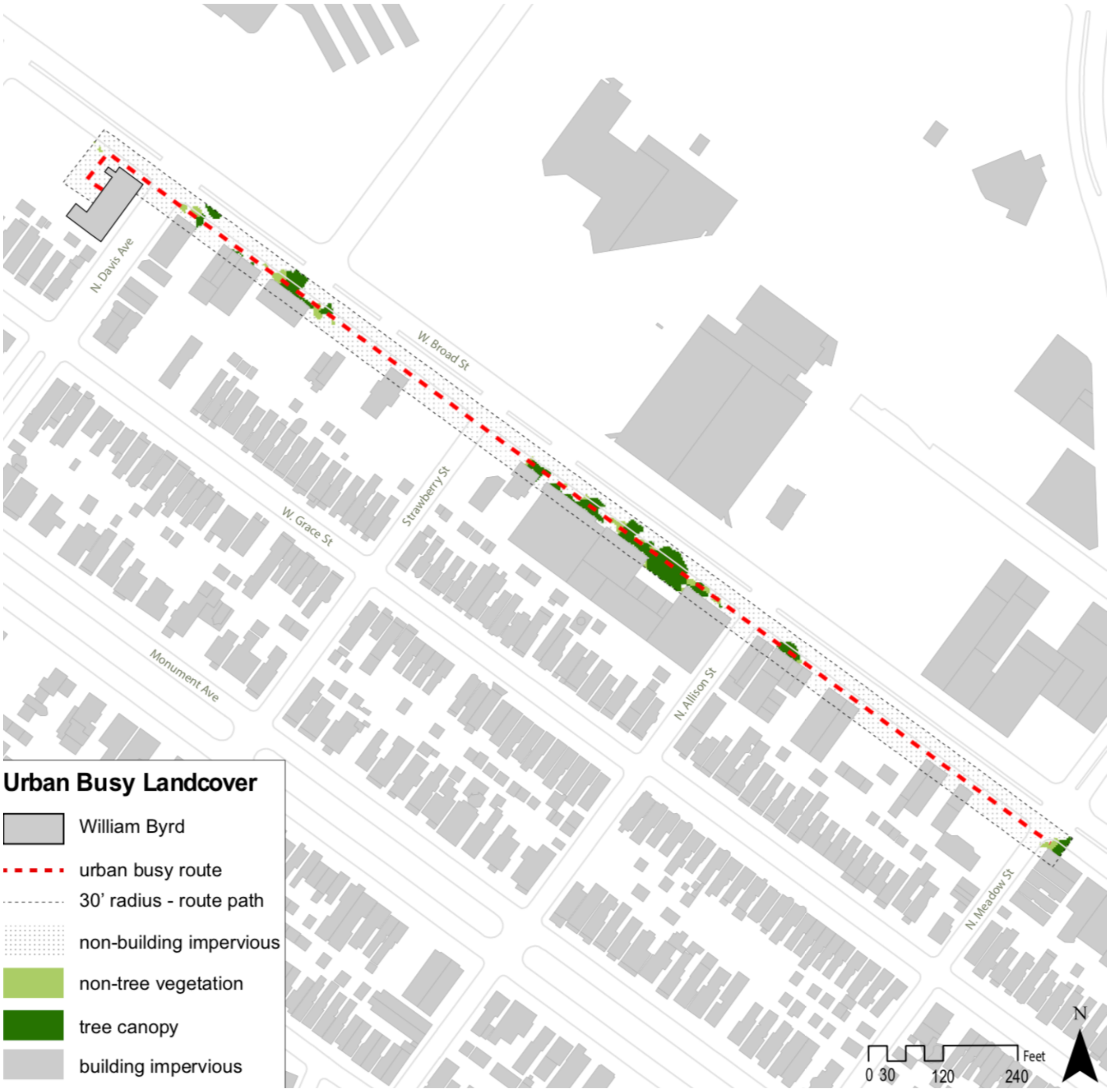

Supplementary Figure 3: air pollution data (PM2.5) Day 1

Note: a hot color (red to yellow) denotes higher PM2.5, a cool color (green) denotes lower PM2.5 levels.

Figure 3 shows that PM2.5 levels started low and consistently increased during the urban gray walk and remained moderately high throughout the urban green walk.

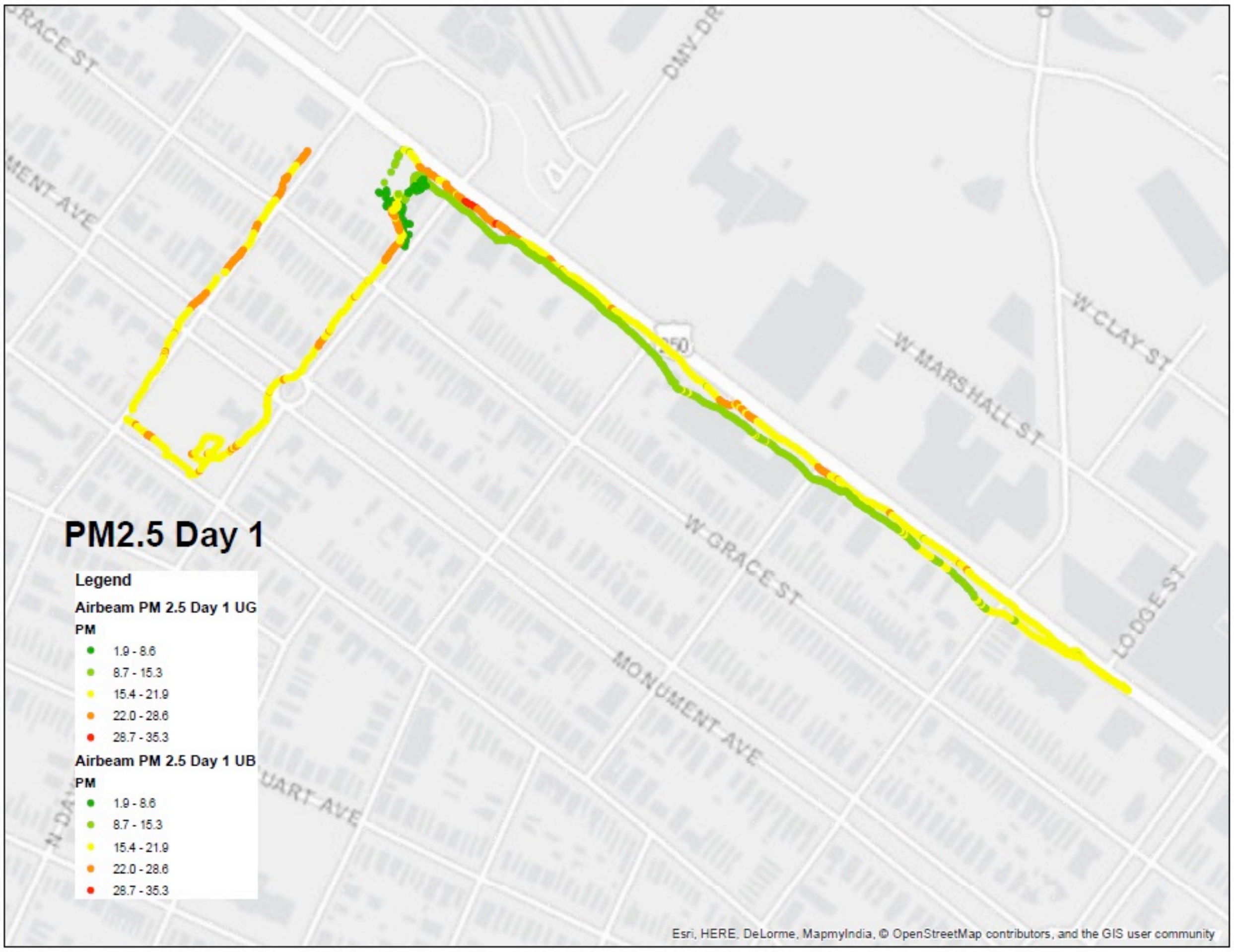

Supplementary Figure 4: **air pollution data (PM2.5), Day 2**

Note: a hot color (red to yellow) denotes higher PM2.5, a cool color (green) denotes lower PM2.5 levels.

Figure 4 shows that PM2.5 levels remained lower on both walks on Day 2.

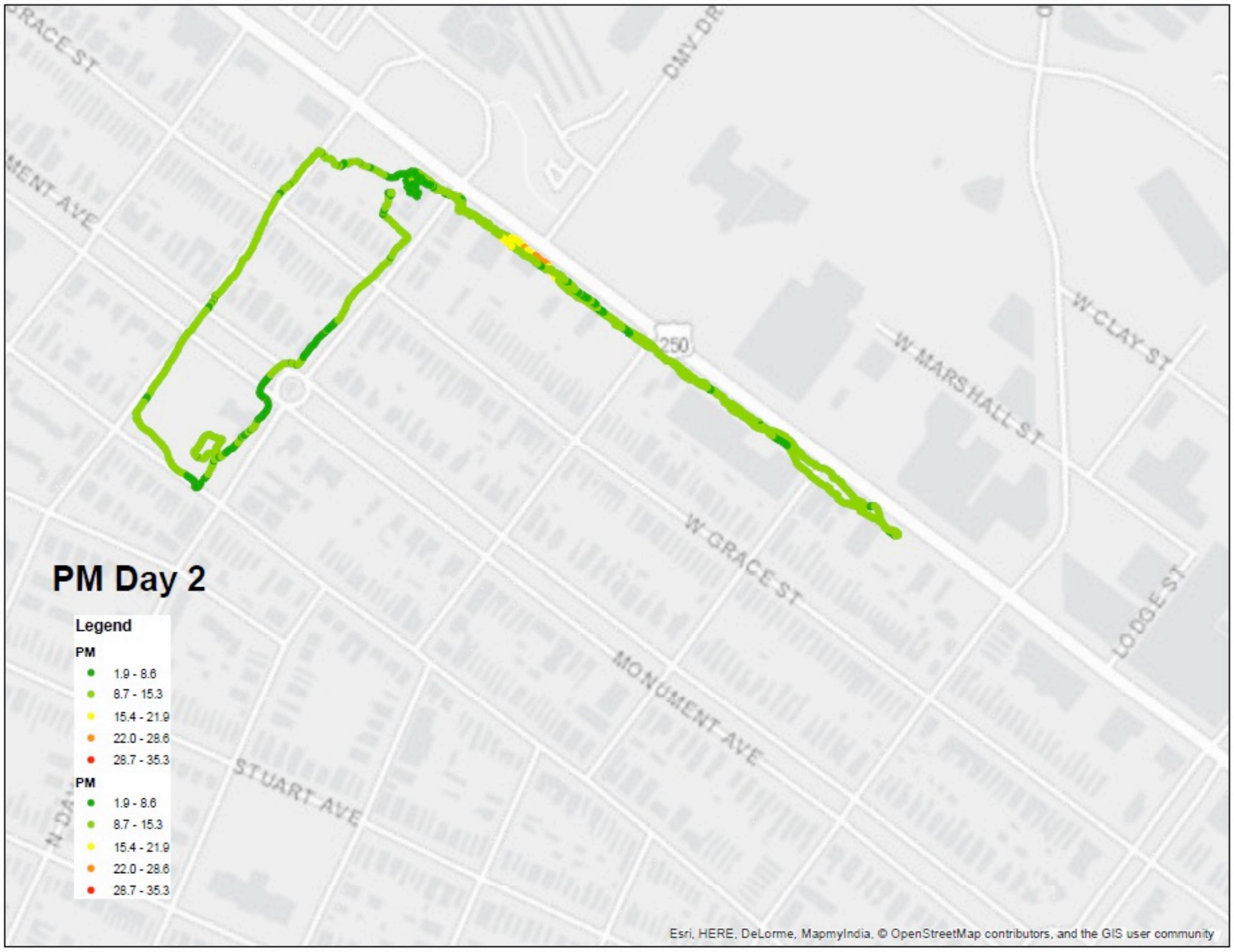

Supplementary Figure 5: **noise** data (dB) Day 1

Note: a hot color (red to yellow) denotes higher dB level, a cool color (green) denotes lower dB levels.

Noise levels are notably higher on the urban gray walk. The urban green walks include several stretches of consistent quietude, with some higher noise levels on the western part of the loop, near Monument Avenue.

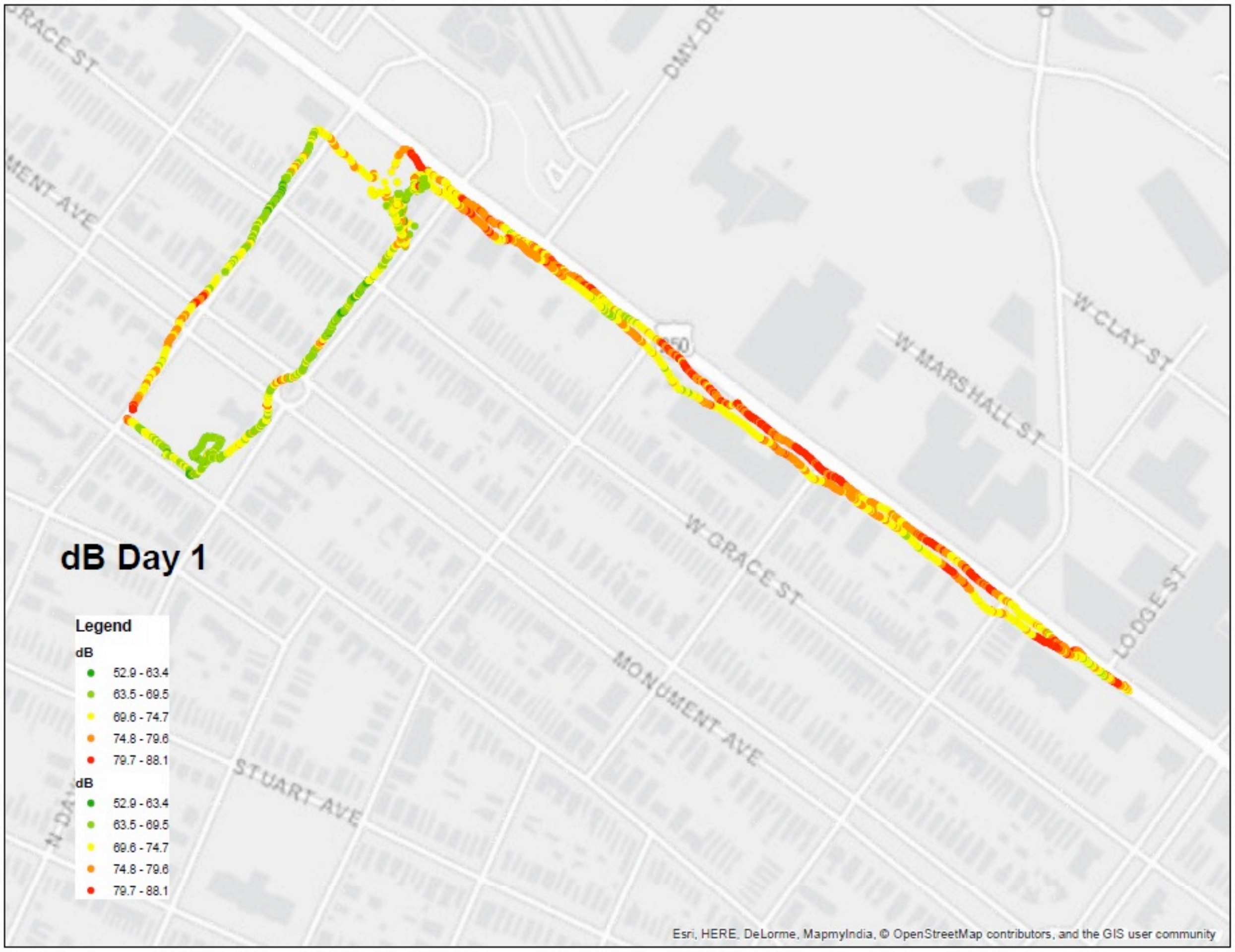

Supplementary Figure 6: noise data (dB) Day 2

Note: a hot color (red to yellow) denotes higher dB level, a cool color (green) denotes lower dB levels.

Noise levels are notably higher on the urban gray walk. The urban green walks include several stretches of consistent quietude, with some higher noise levels on the western part of the loop, near Monument Avenue.

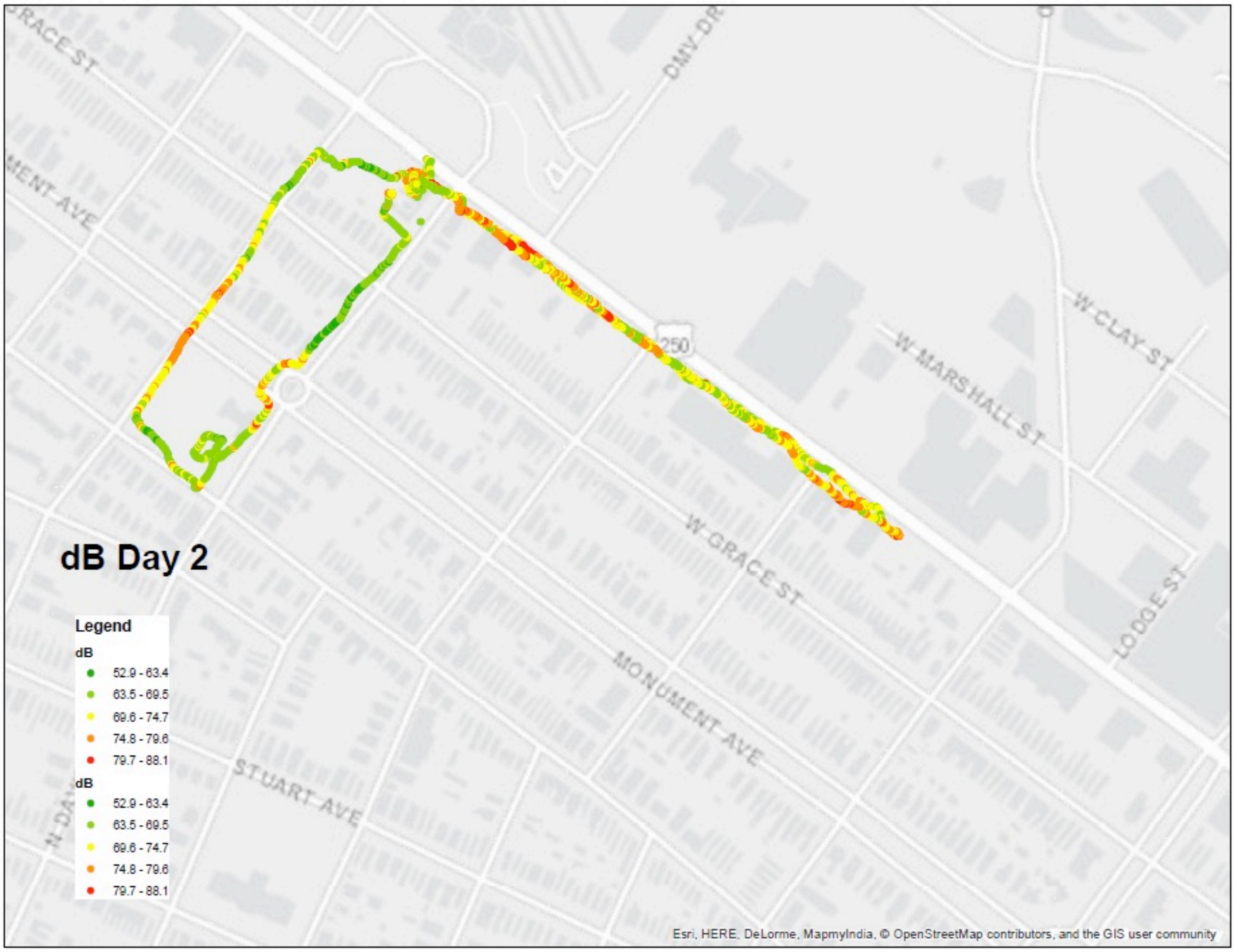

Supplement: Supplementary file 1 [file Data_Sheet_1.pdf]
